# Exposure to lead-free frangible firing emissions containing copper and ultrafine particulates leads to increased oxidative stress in firing range instructors

**DOI:** 10.1186/s12989-022-00471-0

**Published:** 2022-05-15

**Authors:** Ryan J. McNeilly, Jennifer A. Schwanekamp, Logan S. Hyder, John P. Hatch, Brett T. Edwards, Jacob A. Kirsh, Jerimiah M. Jackson, Thomas Jaworek, Mark M. Methner, Christin M. Duran

**Affiliations:** 1 711th Human Performance Wing, Air Force Research Laboratory, Wright Patterson Air Force Base, Dayton, OH USA; 2 Integrative Health and Performance Sciences Division, UES, Inc., Dayton, OH USA; 3 National Institute for Occupational Safety and Health, Cincinnati, OH USA

**Keywords:** Exposure assessment, Biomarkers, Ultrafine particulate, Firing range, Carbon monoxide, Copper, Oxidative stress, Ventilation

## Abstract

**Background:**

Since the introduction of copper based, lead-free frangible (LFF) ammunition to Air Force small arms firing ranges, instructors have reported symptoms including chest tightness, respiratory irritation, and metallic taste. These symptoms have been reported despite measurements determining that instructor exposure does not exceed established occupational exposure limits (OELs). The disconnect between reported symptoms and exposure limits may be due to a limited understanding of LFF firing byproducts and subsequent health effects. A comprehensive characterization of exposure to instructors was completed, including ventilation system evaluation, personal monitoring, symptom tracking, and biomarker analysis, at both a partially enclosed and fully enclosed range.

**Results:**

Instructors reported symptoms more frequently after M4 rifle classes compared to classes firing only the M9 pistol. Ventilation measurements demonstrated that airflow velocities at the firing line were highly variable and often outside established standards at both ranges. Personal breathing zone air monitoring showed exposure to carbon monoxide, ultrafine particulate, and metals. In general, exposure to instructors was higher at the partially enclosed range compared to the fully enclosed range. Copper measured in the breathing zone of instructors, on rare occasions, approached OELs for copper fume (0.1 mg/m^3^). Peak carbon monoxide concentrations were 4–5 times higher at the partially enclosed range compared to the enclosed range and occasionally exceeded the ceiling limit (125 ppm). Biological monitoring showed that lung function was maintained in instructors despite respiratory symptoms. However, urinary oxidative stress biomarkers and urinary copper measurements were increased in instructors compared to control groups.

**Conclusions:**

Consistent with prior work, this study demonstrates that symptoms still occurred despite exposures below OELs. Routine monitoring of symptoms, urinary metals, and oxidative stress biomarkers can help identify instructors who are particularly affected by exposures. These results can assist in guiding protective measures to reduce exposure and protect instructor health. Further, a longitudinal study is needed to determine the long-term health consequences of LFF firing emissions exposure.

**Supplementary Information:**

The online version contains supplementary material available at 10.1186/s12989-022-00471-0.

## Background

In the early 2000s, United States Air Force (USAF) small arms firing ranges began transitioning to copper based, lead-free frangible (LFF) ammunition due to health, safety, and environmental concerns associated with lead-based ammunition [1–4]. Before this widespread transition, a review was conducted of the existing body of knowledge on the health effects of LFF ammunition exposure [5]. Two Army studies investigated toxic emissions from LFF ammunition and lead ammunition with a lead-free primer. Both studies, performed in sealed chambers, found that the level of carbon monoxide (CO) present in weapons emissions was lower using LFF ammunition compared to lead ammunition [5]. The main conclusion was that LFF ammunition is preferred over lead-based ammunition in firing ranges. However, because there is still some risk associated with exposure, efforts should be made to maintain ventilation systems and mitigate exposure to emissions [5].

Despite this switch, multiple USAF studies have shown that firing range instructors continued to report adverse health effects such as sore throat, chest tightness, respiratory and eye irritation, and metallic taste [1, 4]. In 2012, a study investigating emissions during the firing of an M4 rifle and M9 pistol using LFF ammunition at a fully enclosed USAF military base in North Dakota observed symptoms including headache (92%), metallic taste (42%), sore throat (33%), shortness of breath (25%), nasal irritation (25%), and nausea (8%) [4]. These symptoms resolved less than a day after exposure suggesting a direct link between firing exercises and symptoms. In 2014, a Norwegian study found that participants firing LFF ammunition reported more instances of headache (74% v 35%), chills (74% vs 53%), nausea (11% vs 0%), metallic taste (42% vs 29%), cough (90% vs 71%), and shortness of breath (26% vs 12%) compared to exposures from lead-based ammunition [6]. Together, these studies demonstrate that exposure to LFF firing emissions results in higher reported symptoms in instructors when compared to lead ammunition.

Historically, most of the investigations into the cause of symptoms have focused on evaluating firing range ventilation systems and measuring copper (Cu) levels in the breathing zone of instructors. The standard for firing range ventilation was developed in 1975 by the National Institute for Occupational Safety and Health (NIOSH) [7]. The Department of Defense (DoD) guidance references these criteria and recommends an air velocity of 0.381 ± 0.0762 m/s (75 ± 15 feet per minute (fpm)) with even distribution across the firing line [8]. However, this criterion is occasionally difficult to achieve in some firing ranges. For example, one study at a USAF facility in Louisiana showed a large variability in air velocity magnitude and direction across multiple firing lanes. Using a fog machine, airflow patterns were observed to reverse direction and occasionally stagnate at or behind the firing line, thereby increasing instructor exposure [1]. Finally, a study conducted at a USAF small arms firing range in Ohio utilized both qualitative and quantitative airflow measurements to assess firing range ventilation. Not only was air velocity highly variable across shooting stalls when the range was fully operational (40–140 fpm), but qualitative fog machine testing showed stagnant air or even backflow at the firing line in several stalls [9]. These studies highlight that both air velocity measurements and qualitative fog measurements are necessary to assess proper range ventilation operation.

The purpose of adequate ventilation is to remove weapons emissions from the facility to maintain air contaminant levels below occupational exposure limits (OELs), and, in turn, reduce exposure. OELs originating from an enforceable standard set by the Occupational Safety and Health Administration (OSHA) are referred to as permissible exposure limits (PELs). Recommended OELs are generated by NIOSH or the American Conference of Governmental Industrial Hygienists (ACGIH) and are typically more conservative. NIOSH and ACGIH standards are updated more frequently to remain current with research efforts; however, they do not take into account technical and financial feasibility. The USAF policy governing OELs requires the use of the most conservative standard from the OSHA, ACGIH, or AF-specific standard [10]. The most conservative OELs for each chemical are utilized in this work and are based on an 8-h time weighted average (8 h TWA), the maximum allowable exposure for an 8 h workday within a 40 h workweek, or a ceiling limit, which is the maximum exposure to which an individual could be exposed for any length of time. The ceiling limit may be directly identified by an organization or estimated using the ACGIH “3/5 rule,” a rule of thumb that the ceiling limit should be no more than five-fold the 8 h TWA limit [11]. Unfortunately, these limits rarely take into consideration exposures to complex mixtures, as is the case with LFF firing emissions.

Over the past decade, several groups have undertaken efforts to characterize LFF firing emissions [1, 4, 6, 9, 12]. LFF ammunition is composed of a primer, propellant, projectile core, and a jacket, all of which can produce emission byproducts upon combustion. Studies internationally and in the USAF determined that emissions from weapons firing LFF ammunition include a complex mixture of metal-containing particulate matter and combustion gases, including CO, carbon dioxide (CO_2_), ammonia (NH_3_), formaldehyde, hydrogen chloride (HCl) and hydrogen cyanide (HCN) [1, 6, 13]. The majority of these gases were detectible in quantities well below OELs, although CO was most abundant in firing emissions [1]. In a NIOSH report from 2013, average CO concentrations in the breathing zone of range instructors during firing events were 8 ppm with a peak concentration of 23 ppm, well below the OSHA 8 h TWA PEL of 50 ppm and also below the more stringent ACGIH OEL of 25 ppm [4].

Studies measuring a wide range of metals confirmed that Cu and zinc were the metal components in highest abundance in firing emissions, with trace amounts of lead, tin, and bismuth [4, 6]. At two independent USAF small arms firing ranges in Colorado and Tennessee, Cu levels (0.0039 mg/m^3^ and 0.055 mg/m^3^, respectively) fell below an 8 h TWA of 0.1 mg/m^3^, the current OSHA PEL for Cu fume. A report from 2008 characterizing Cu emissions in the breathing zone of range instructors found that two of the five bases studied had 8 h TWA Cu levels that were above (0.2 mg/m^3^, Missouri) or at (0.1 mg/m^3^, South Carolina) the OSHA regulated PEL [1]. However, more recent reports observed that total Cu levels measured in the breathing zone of instructors fell below the OSHA PEL for Cu fume (0.026 mg/m^3^ and 0.005 mg/m^3^ respectively). Cu 8 h TWA measurements have never exceeded the OSHA PEL for Cu dust (1.0 mg/m^3^) [4, 9].

Because mass-based OELs have not been frequently exceeded, recent studies have shifted focus to further investigate particulate matter as a function of particle size. A Norwegian study utilizing an enclosed firing chamber observed that particulate matter concentrations were higher when firing LFF ammunition compared to lead-based ammunition [6]. Previous work has shown that particles emitted during small arms firing are in the ultrafine size range (< 100 nm), and that particle number concentration in the breathing zone increased during firing events [14]. During shooting events with LFF ammunition in a fully enclosed USAF firing range, particulates measured in an area sample showed median diameters ranging from 40–80 nm [14] which classified these particles as ultrafine [15, 16]. In a controlled chamber environment, ultrafine particles (UFP) contributed most to number density immediately following firing where > 90% of particles were < 30 nm in diameter [12, 17].

Due to their small size, UFPs are able to deposit in the alveolar space of the lungs, bypassing the normal defenses against inhaled particles (mucociliary clearance), where they can cause the development of respiratory diseases and/or the exacerbation of existing diseases [15, 16]. This region of the lungs is involved in gas exchange and, here, UFPs can translocate into the bloodstream where they can impact other organs including the brain and heart leading to the development of neurodegenerative and cardiovascular diseases [15, 18, 19]. Additionally, UFPs can damage lung epithelial cells by inducing reactive oxygen species (ROS) production which can cause DNA damage, among other negative effects [18]. In fact, the primary mechanisms of UFP toxicology are thought to be induction of the inflammatory and oxidative stress responses [18]. Together these data suggest that UFP exposure may be contributing to symptoms experienced by range instructors. However, a relevant exposure standard for UFPs in firing emissions does not exist. UFP contribution to mass-based measurements is negligible and, therefore, mass-based OELs may not be appropriate [20].

Under normal conditions, antioxidants and oxidative stress are maintained in a balanced dynamic in the body. Upon introducing a stressor, such as inhaled UFPs, there is an increase in ROS production which causes DNA damage. Specifically, the nucleotide deoxyguanosine is hydroxylated on carbon at position 8 creating 8-hydroxydeoxyguanosine (8-OHdG). This initiates the DNA damage repair pathway resulting in the excision of 8-OHdG which is excreted in measurable quantities in urine [21]. In fact, 8-OHdG is a common biomarker used to assess oxidative DNA damage [22–24].

To date, there have been no studies investigating LFF firing emissions where exposure monitoring of instructors, measurements of biomarkers of exposure and effect, and documentation of symptoms before and after training classes are combined for a comprehensive exposure assessment. In a study where total Cu and dust levels exceeded ACGIH threshold limit values by a factor of 1.7 and 30 respectively when shooting a steel core lead-free ammunition compared to lead-based, no decline in lung function was observed despite participants reporting symptoms including headache, coughing, and shortness of breath [6]. However, these are the only biological and physiological indices measured in this study. Biofluids were not collected and analyzed for differences in metals and biological markers of stress from lead-free ammunition compared to lead-based. Therefore, a clear picture of how exposure to LFF firing emissions physiologically impacts the body remains unknown.

Although few studies have looked at biomarkers of exposure in firing range instructors, a study from Belgium did find that lead was elevated in blood and urine of firing range employees, including instructors, compared to a control group [25]. Other occupations where exposure to heavy metals is common may also provide insight into the biological response to firing emissions. For example, a study investigating exposures to automobile welders showed that heavy metals (Cu, cadmium, zinc, and lead) were detectible at higher levels in the urine of welders when compared to office workers, suggesting that urinary metal concentration may be a viable method to monitor inhaled metal exposure [26].

Currently, the exposure assessment paradigm in USAF small arms firing ranges consists of breathing zone air monitoring and ventilation measurements. This paradigm does not take into consideration the biological burden of LFF firing emissions exposure. Such a mixture makes it difficult to understand and explain why instructors continue to report adverse symptoms, despite OELs not being exceeded. The ability to correlate exposure with symptoms and health consequences is essential for driving recommendations to improve health and safety. To this end, noninvasive measurements of biomarkers of exposure and effect may aid the development of new monitoring technologies that can help identify hazards during exposure assessment activities.

The study presented here will address two main gaps in understanding by: (1) Determining the exposure of firing range instructors to metals, combustion gases, and UFPs and (2) Using biomarkers to assess range instructor exposure and the physiological consequences of exposure as well as epidemiological questionnaires to document symptoms. To do this, firing emissions exposure was investigated at both a fully enclosed range and a partially enclosed range. Ventilation measurements were completed at both ranges to assess air velocity and direction in comparison to the AF standard. Additionally, firing emissions were measured in the breathing zone of instructors using a suite of real-time monitoring instruments and offline methods.

Lung function, urinary 8-OHdG, and urinary Cu were measured before and after each work shift. Additionally, epidemiological questionnaires captured symptoms and lifestyle exposures that may impact biological measurements. Biomarker data were compared with emissions data and questionnaire outcomes to determine a method to monitor range instructors for exposure and to support updates to exposure standards. Finally, observational studies were utilized to correlate peaks in exposure to biomarkers of oxidative stress along with symptoms reported by range instructors to obtain a more complete picture of exposure.

## Results

### Air velocity and direction at the firing line at both Wright Patterson Air Force Base (WPAFB) and Joint Base Charleston (JBC) were highly variable

**Fig. 1:**
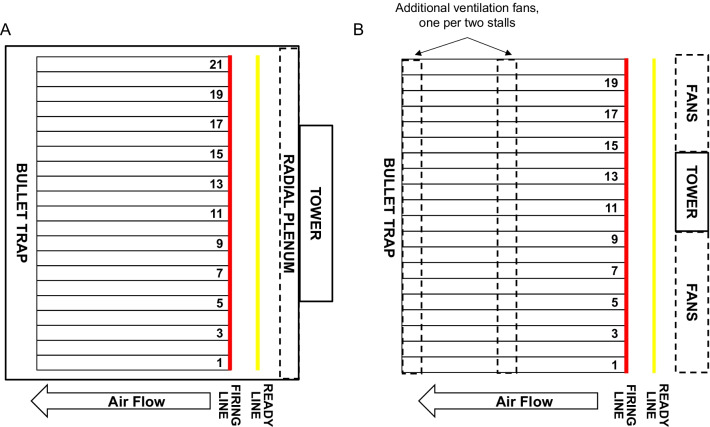
Schematics of small arms ranges at WPAFB and JBC. **A** Illustration of the fully enclosed range design at WPAFB where ventilation is supplied by a radial plenum. **B** Illustration of the partially enclosed range design at JBC where ventilation is provided by banks of industrial fans

**Fig. 2:**
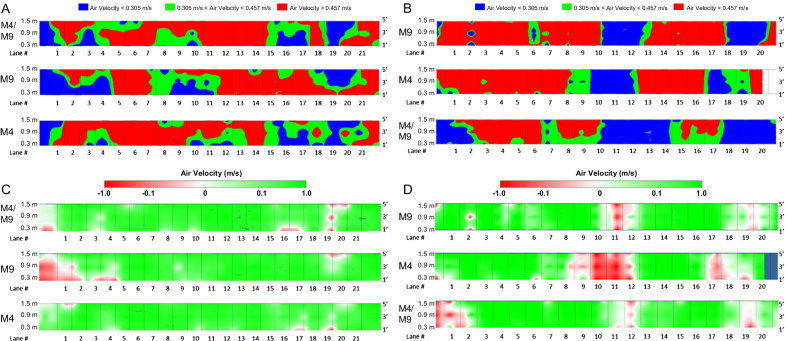
Air flow velocities and direction at firing line for classes at WPAFB and JBC. **A** Air velocity measured over three days at WPAFB for the indicated class types. **B** Air velocity measured at JBC for the indicated class types. **C** Directional air flow velocities measured at WPAFB. **D** Directional air flow velocities measured at JBC. Measurements were made at each base prior to or after each of the M9. M4, and M4/M9 classes

WPAFB and JBC were selected for this study due to the differing range designs and ventilation systems. WPAFB is a fully enclosed range with ventilation provided by a push–pull system with a perforated radial-style air delivery plenum with exhaust and air return ducts at the bullet trap (Fig. 1A) [27]. JBC is a partially enclosed range (roof and sidewalls) with the back of the range open to the outdoors and three banks of overhead industrial fans for ventilation (Fig. 1B). Flow maps of the nine-point ventilation measurements made within each shooting stall at WPAFB and JBC showed that neither of the ranges had consistent airflow across all the stalls (Fig. 2A, B). Average flow velocities exceeding the recommended upper limit of 0.457 m/s (90 fpm) were observed across all stalls on each day of testing at both bases (Fig. 2A, B, red). At WPAFB, low airflow was consistently observed to the left of stall one and in stalls 3, 4, and 19 (Fig. 2A, blue). Additionally, lanes 12 through 14 consistently registered higher flow velocities (Fig. 2A, red). It is important to note that ventilation fans at JBC flanked either side of the fire control tower but were not present above or behind the tower (Fig. 1B). Lanes 10–14 were positioned directly in front of the tower and displayed reduced flow (Fig. 2B). Low flow was also observed in lanes 17–20 due to the fact that fans supplying these lanes were inoperable at the time of data collection. The remaining lanes had air velocities above 0.457 m/s (90 fpm) (Fig. 2B, red).

Under optimal conditions, airflow should proceed from the firing line downrange to the bullet trap thereby carrying emissions away from students and instructors. However, at both WPAFB and JBC, regions of negative airflow were observed (Fig. 2C, D, Additional file 1: Fig. S1 and Additional file 2: Fig. S2). This occurred primarily in stalls nearest the walls of the range at WPAFB (Fig. 2C, red) and in front of the tower at JBC (Fig. 2D, red). Additionally, negative airflow was detected in lanes 17–20 at JBC, corresponding to ventilation fans that were inoperable (Fig. 2D, red). Interestingly, areas of low flow (Fig. 2A, B, blue) corresponded to red and white areas of negative and stagnant airflow, respectively (Fig. 2C, D). This was qualitatively determined by the use of a fog machine (Additional file 1: Fig. S1 and Additional file 2: Fig. S2).

Wind speed and direction data were collected during the M4 and M4/M9 classes at JBC (Additional file 3: Fig. S3). Over the sampling period, the prevailing winds blew from the southern direction, particularly on either side of the tower at speeds equal to or below 2 m/s. This is consistent with local meteorological data where winds during the sampling week blew primarily from the South, although wind speeds were higher (2.84 to 5.81 m/s) than those measured in range [28]. Stronger winds with more variability from the southern direction were observed during the M4/M9 class (Additional file 3: Fig. S3A). Wind direction during the M4 class was primarily from the south, with some winds from the South East on the right side of the range (Additional file 3: Fig. S3 B). Winds measured on the right side of the range, where two fans were inoperable, were more variable.

### Demographic information associated with study population

In total, 21 Air Force Security Force personnel were recruited for this study between both WPAFB and JBC (Table 1). Range instructors were exposed to LFF firing emissions as part of their daily job responsibilities, while the control group was not exposed to emissions. The 10 control subjects typically worked in an office or on patrol in a squad car (Additional file 4: Table S1). The combined range instructor population across both bases was primarily male and all identified as Caucasian with an average age of 29. However, instructors at JBC were significantly older (31–35 y) than their WPAFB counterparts (23–34 y) (Table 1).

**Table 1:** Select demographics by base and participant group

Variables	*WPAFB*			*JBC*		
	All *n* = 12	Instructors *n* = 7	Controls *n* = 5	All *n* = 9	Instructors *n* = 4	Controls *n* = 5
Male^+^	83.3%	85.7%	80.0%	55.6%	100%	20.0%
Caucasian	100%	100%	100%	66.7%	100%	40.0%
Age (y)^++^ ± SD	25.8 ± 3.76	27.4 ± 3.46	23.4 ± 3.05	28.1 ± 4.51	32.5 ± 1.73	24.6 ± 1.95
BMI ± SD	25.9 ± 3.49	26.5 ± 3.94	25.21 ± 3.00	25.4 ± 3.59	26.6 ± 4.57	24.5 ± 2.77
E-5 Rank	50.0%	57.1%	40.0%	77.8%	75.0%	80.0%
Years at Base ± SD	1.5 ± 1.6	1.6 ± 1.9	1.5 ± 1.3	2.4 ± 1.9	2.0 ± 1.8	2.7 ± 2.0

+Sex of JBC instructors versus JBC controls (*p* = 0.0476)

++All instructors versus all controls (*p* = 0.0015); Instructors at WPAFB versus JBC instructors (*p* = 0.0243)

SD, Standard Deviation; n, number of participants; All, Instructors + controls; BMI, body mass index. E-5 Rank corresponds to the rank of Staff Sergeant in the USAF

The control population across WPAFB and JBC was 50% female and the majority identified as Caucasian with an average age of 24 (age range 20–27 y). The instructor group, overall, was significantly older than the control group (Table 1).

Daily pre-shift questionnaires asked subjects about their engagement in certain activities outside of their work shifts due to their potential to produce symptoms similar to those of interest in this study (Table 2). More WPAFB instructors reported recreational shooting than the other subject groups, reporting an average of two additional hours and 170 rounds per week. Other off-duty hobbies were reported too infrequently to assess their potential impact on symptom reports. In addition to external activities, health behavior data was also collected. Use of tobacco products was more prevalent among WPAFB subjects than JBC subjects, and WPAFB controls consumed less caffeine than other subject groups, who all used it daily (Table 2).

**Table 2:** Lifestyle factors of interest by base and participant group

Variables	*WPAFB*			*JBC*		
	All *n* = 12	Instructors *n* = 7	Controls *n* = 5	All *n* = 9	Instructors *n* = 4	Controls *n* = 5
Tobacco Use	33.3%	42.9%	20.0%	11.1%	25.0	0.00%
Caffeine Use^+^	75.0%	100%	40.0%	100%	100%	100%
Dietary Supplements	41.7%	28.6%	60.0%	44.4%	75.0%	20.0%
Prescription Steroids	8.30%	14.3%	0.00%	0.00%	0.00%	0.00%
Allergies	25.0%	28.6%	20.0%	44.4%	25.0%	60.0%
Recent Illness	16.7%	14.3%	20.0%	11.1%	25.0%	0.00%
Recreational Shooting	50.0%	71.4%	20.0%	11.1%	0.00%	20.0%

^+^Caffeine use WPAFB instructors versus WPAFB controls (*p* = 0.046)

SD, standard deviation; n, number of participants; All, Instructors + controls

Data on seasonal, pet, or environmental allergies were also collected among the other lifestyle factors. Allergies were more prevalent in JBC subjects than in WPAFB subjects. Among the 7 individuals across both bases with allergies, only 42.9% reported that their allergy symptoms were completely controlled by medication. However, allergies were not correlated with symptom outcomes.

### Firing range class duration and rounds fired

Eight classes were assessed at WPAFB versus four classes at JBC. The hours spent on the line per weapon and class by range instructors did not differ significantly between bases (Table 3). The average class size at JBC was significantly larger than that of WPAFB (18.3 ± 0.96 vs 8.25 ± 3.77, *p* < 0.001) (Table 3). Classes from both bases fired approximately the same number of rounds per student for both the M9 (90 rounds) and M4 (206–286 rounds) with the exception of 07 July 19 at JBC where the M4 class was canceled due to weather after firing 8 rounds. The total number of rounds fired during assessments at WPAFB were approximately 4200 rounds of M9 and 9200 rounds of M4. The total number of rounds fired during assessments at JBC were approximately 3200 rounds of M9 and 9000 rounds of M4.

**Table 3:** Range training class information for instructors by base

Per Class	WPAFB (mean ± SEM)	JBC (mean ± SEM)
Class Size	8.25 ± 1.33	18.25 ± 0.48*
Time on the line, M9 (h)	1.55 ± 0.95	1.3^+^
Time on the line, M4 (h)	2.75 ± 0.05	3.1^+#^
Time on the line, M9/M4 (h)	2.70 ± 0.62	6.0^+^

^+^No standard error of the mean (SEM); only a single day of measurement

^#^Class from 07 Jul 19 excluded due to cancelation after 30 min

*p < 0.001 v WPAFB

### Range instructors reported significantly more adverse symptoms compared to control participants

Range instructors at both WPAFB and JBC reported experiencing a variety of symptoms during the data collection period while only one control subject reported symptoms of a headache (Table 4). Being a range instructor was significantly correlated with reporting post-shift symptoms during the study period (*p* < 0.0001). Thus, only symptoms reported by instructors were examined further.

**Table 4:** Symptoms reported by range instructors on post-shift questionnaires per base

Symptoms,	Combined *n* = 50	WPAFB *n* = 31	JBC *n* = 19
Chest Tightness	18.0%	16.1%	21.1%
Metallic Taste	18.0%	22.6%	10.5%
Sore/Scratchy Throat	16.0%	9.70%	26.3%
Itchy/Watery Eyes^+^	12.0%	6.50%	21.1%
Headache^++^	10.0%	12.9%	5.30%
Cough	10.0%	6.50%	15.8%
Nausea/Appetite Loss	10.0%	16.1%	0.00%
Breathing Trouble	8.00%	9.70%	5.30%
Runny/Itchy/Congested Nose	4.00%	3.20%	5.30%
Chills/aches	2.00%	0.00%	5.30%
Dizziness	2.00%	0.00%	5.30%
Wheezing	2.00%	0.00%	5.30%

^+^Post-shift eye irritation was reported significantly more at JBC than WPAFB (*p* = 0.0454)

^++^Three (14%) questionnaires from a JBC control indicated headache. No other symptoms were reported by control groups

On the initial questionnaire, 54.5% of all range instructors reported experiencing one or more symptoms during or within 24 h of exposure to LFF firing emissions in the past month. Questionnaires asked subjects to select as many symptoms as were applicable from a list of 12 options. Each base reported a unique profile of historical symptoms. Among those who reported symptoms in the past month, JBC instructors most commonly experienced itchy or watery eyes (100%) followed equally by cough, trouble breathing, chest tightness, and headache (66.6%). WPAFB instructors reported historically experiencing nausea or appetite loss and metallic taste at equal frequencies (100%) followed by trouble breathing (66.6%).

Over the study period, 50 post-shift questionnaires were collected from range instructors. Instructor attendance and length of the assessment period at each base did not significantly impact the number of post-questionnaires collected. Among all instructors, 63.6% reported experiencing one or more symptoms during a shift at least once on a post-questionnaire (Table 4). The most frequently reported symptom on post-questionnaires was sore or scratchy throat at JBC, which did not mirror initial questionnaire reports, and metallic taste at WPAFB, which was similar to initial questionnaire reports (Table 4). No significant correlations were observed between total symptoms reported and demographic or lifestyle factors; however, these trends could change with longer observation periods and larger sample sizes.

Of note, a single individual at each base contributed a large portion of the total post-shift symptoms reported. Forty percent of JBC post-shift questionnaires that contained symptom reports were submitted by one individual while 33.3% of WPAFB post-shift questionnaires that contained symptom reports were submitted by one individual. However, the individual at JBC was on the firing line every day during our study whereas the WPAFB individual was never on the firing line during the study period. Furthermore, the difference in average total symptoms reported by instructors on the firing line during JBC classes versus WPAFB classes was nearly significant (*p* = 0.056). There was a positive correlation between total daily symptoms reported and hours spent on the line per class (*p* = 0.026), weapon type (*p* = 0.026), and total rounds fired per student (*p* = 0.022) respectively. This suggests a possible dose–response relationship between certain class characteristics and acute health outcomes in range instructors after exposure to LFF firing emissions.

### Airborne Cu particles were increased in breathing zone of range instructors during firing range classes

**Fig. 3:**
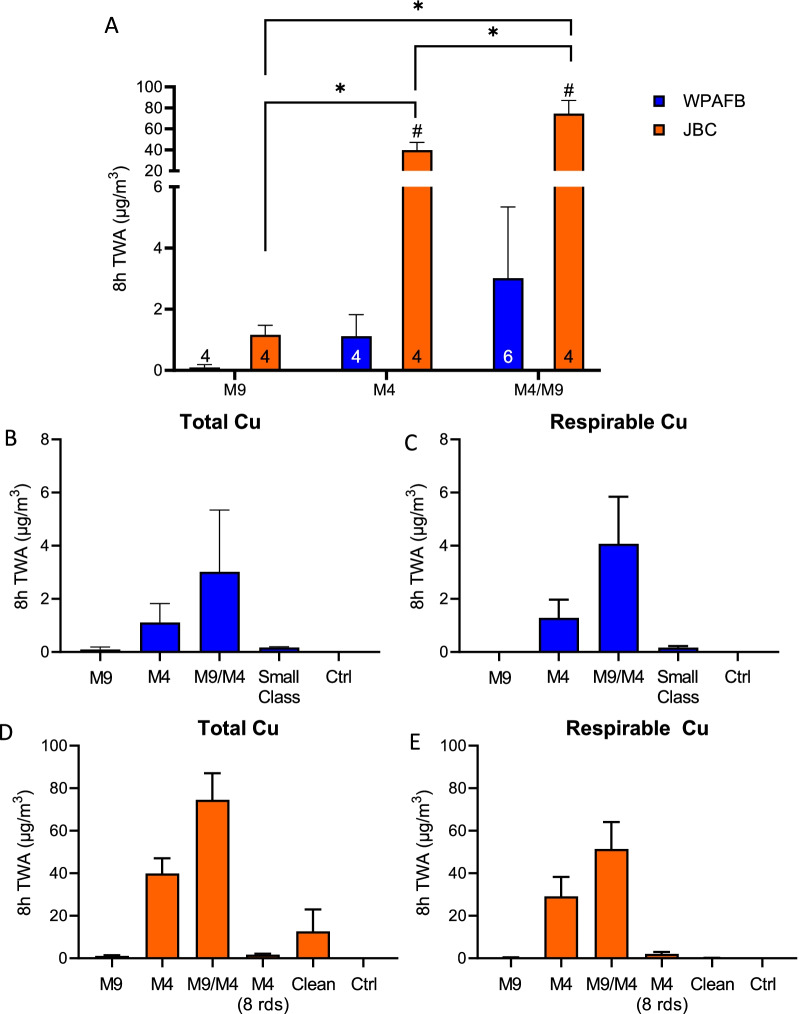
Copper exposure measured in the breathing zone of instructors. **A** Average 8 h TWA Cu measurements at both WPAFB and JBC based on class type. Not including canceled M4 class. Error bars represent SEM. *p < 0.01. #*p* < 0.01 versus WPAFB. n shown above x-axis. **B** Total copper measurements collected at WPAFB. **C** Cu measured in the respirable range at WPAFB. **D** Total Cu measurements collected at JBC. **E** Cu measured in the respirable range at JBC. Error bars represent SEM. “Small Class” represents a class size of less than 5 students and contains data from both M4 and M9 refire events. 8 rds = 8 rounds of ammunition fired. “Clean” indicates a day with no class at JBC in which range instructors were cleaning the range

Airborne Cu particulate remains the air quality safety standard utilized for firing ranges in the AF and was measured in this study. Cu exposure varied between the bases and by class type. Overall, Cu measurements were higher at JBC than at WPAFB. The highest total Cu exposure was measured during the combined M4/M9 class for both WPAFB and JBC, with average 8 h TWA values of 3.01 µg/m^3^ and 74.6 µg/m^3^, respectively (Fig. 3A). In fact, total Cu levels measured at JBC exceeded the action level (50 µg/m^3^) for Cu fume during the M4/M9 class. Although M9 firing had far lower exposure compared to other classes for both ranges, JBC still had significantly higher exposure compared to WPAFB (1.15 µg/m^3^ vs 0.093 µg/m^3^) (Fig. 3A). Additionally, the M4/M9 class at JBC created a significantly higher exposure (74.6 µg/m^3^) than the M4 (39.8 µg/m^3^) or M9 (1.15 µg/m^3^) classes alone (Fig. 3A). No significant differences in exposure between weapon types was detected at WPAFB.

To further investigate exposure, the respirable fraction of Cu was also evaluated and compared to total Cu for each class type. At both WPAFB and JBC, the majority of Cu was in the respirable range (Fig. 3B vs C and D vs E). When considering the M4 and M4/M9 classes at JBC, the average 8 h TWA for the respirable Cu fraction contributed approximately 73.1% (29.1 µg/m^3^ respirable, 39.8 µg/m^3^ total) and 69.0% (51.4 µg/m^3^ respirable, 74.6 µg/m^3^ total) to the total Cu, respectively (Fig. 3D, E). Interestingly, small amounts of M4 firing (n < 5 participants at WPAFB, only 8 rounds at JBC) produced more Cu exposure than their respective full M9 classes (Fig. 3B, D).

Finally, the abbreviated M4 class at JBC where only 8 rounds of ammunition (n = 19 students, 0.5 h) were fired produced similar total Cu exposure to the full M4 class (n = 9–10 students, 2.7 h) at WPAFB (WPAFB M4–1.11 µg/m^3^, JBC M4 8 rds–1.68 µg/m^3^, Fig. 3B v D). Additionally, the instructors at JBC did receive some exposure during normal cleaning duties. However, the respirable Cu fraction was minimal, suggesting that most of the exposure was from larger Cu particles (Fig. 3D vs E, clean). Cu was not detected in samples collected in the breathing zone of control participants.

Analysis of particles collected in the breathing zone also indicated exposure to several other metals (Additional file 5: Table S2). These results demonstrated similar trends to Cu measurements, with the highest exposure for most metals occurring during the combined M4/M9 class. Of the metals measured, potassium, zinc, and tin had the highest airborne concentrations, although none approached their respective exposure limits (Additional file 5: Table S2).

### CO and UFPs were increased in the breathing zone of range instructors during firing range classes

**Fig. 4:**
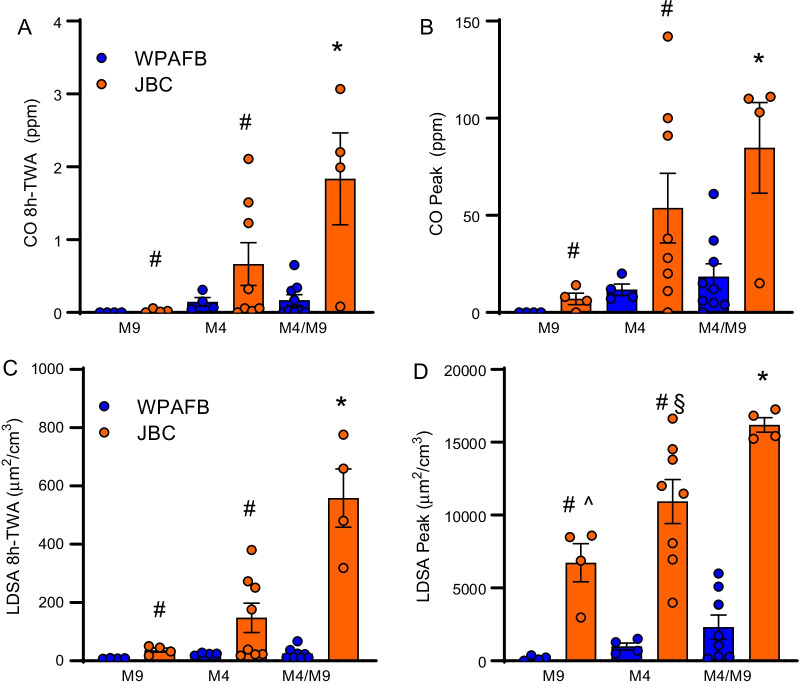
CO and particle exposure based on class type. **A** Average instructor CO 8 h TWA by class type. **B** Peak CO 8 h TWA by class type. **C** Average instructor particle concentration LDSA 8 h TWA by class type **D** Peak LDSA by class type. Data are represented individual values along with bars representing the mean ± SEM. *p < 0.05 JBC versus all WPAFB. #*p* < 0.05 v JBC M4/M9. ^*p* < 0.05 v WPAFB M9. §*p* < 0.05 versus WPAFB M4

The 8 h TWA CO levels observed at JBC were significantly higher during the M4/M9 class (1.84 ppm) when compared to other classes at both JBC (M4 = 0.66 ppm, M9 = 0.023 ppm) and WPAFB (M4/M9 = 0.17 ppm, M4 = 0.15 ppm, and M9 = 0 ppm) (Fig. 4A). Overall, JBC instructors experienced significantly higher levels of CO exposure compared to instructors at WPAFB although neither base approached the ACGIH 8 h TWA limit for CO of 25 ppm. These same trends were observed when evaluating peak CO levels measured at both bases where CO exposure was higher at JBC compared to WPAFB (Fig. 4B). M4/M9 firing at JBC produced significantly higher average peak CO levels (84.7 ppm) than M9 firing at JBC (7.0 ppm) and all of the classes at WPAFB (M4/M9 = 18.4 ppm, M4 = 11.8 ppm, and M9 = 0 ppm) (Fig. 4B). Peak CO levels at JBC did approach the ACGIH recommended ceiling limit of 125 ppm during the M4/M9 (three of four instructors saw peaks above 100 ppm) and was surpassed during M4 firing (highest peak of 142 ppm) (Fig. 4B).

UFP exposure was monitored using lung deposited surface area (LDSA) in µm^2^/cm^3^ for both bases and showed similar trends to CO and Cu exposure, with JBC instructors experiencing significantly higher levels of UFP exposure compared to instructors at WPAFB for all class types (Fig. 4C, D). The most appreciable difference in LDSA 8 h TWA was observed during the combined M4/M9 class where JBC had significantly higher exposure compared to WPAFB (559 µm^2^/cm^3^ vs 23 µm^2^/cm^3^) (Fig. 4C). Additionally, LDSA measurements at JBC during the M4/M9 class were significantly higher than both the M4 (148 µm^2^/cm^3^) and M9 (36 µm^2^/cm^3^) classes (Fig. 4C). At JBC, the highest peak exposure was detected during the M4/M9 class (16,200 µm^2^/cm^3^) compared to M4 (10,900 µm^2^/cm^3^) and M9 (6,730 µm^2^/cm^3^) (Fig. 4D). Interestingly, the M9 class at JBC, which had the lowest exposure, was still higher than the highest exposure at WPAFB which was during the M4/M9 class for both peak (6730 µm^2^/cm^3^ vs 2170 µm^2^/cm^3^) (Fig. 4D) and 8-h TWA (36.4 µm^2^/cm^3^ vs 23.4 µm^2^/cm^3^) (Fig. 4C).

**Fig. 5:**
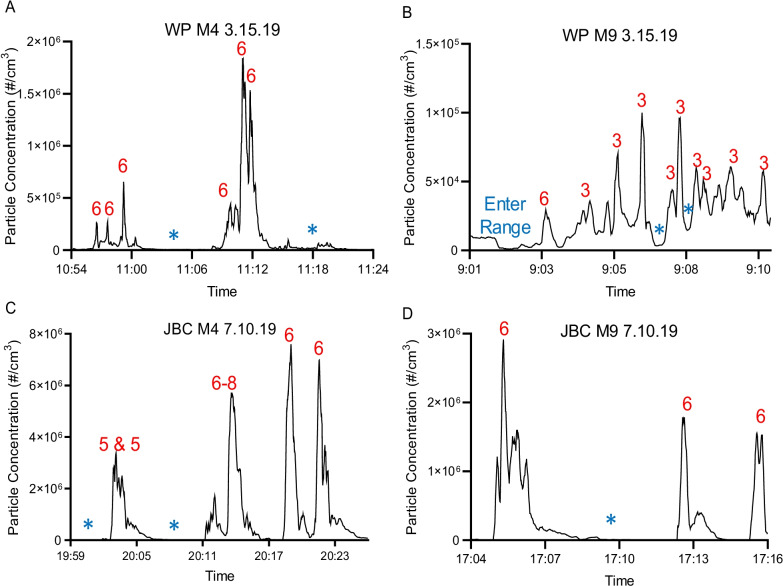
Representative time series plots for average particle concentration. Data are presented as average particle concentration for all of the instructors on the line during the M4/M9 combined classes on 15 MAR 19 at WPAFB and 10 JUL 19 at JBC. **A** WPAFB M4; **B** WPAFB M9; **C** JBC M4; **D** JBC M9. Red numbers above peaks represent the number of rounds fired by each shooter in the class. Nine shooters were present at WPAFB and seventeen at JBC. Blue stars represent times where firing was paused due to grading, instruction, or moving barricades

In order to show that peaks in particle concentration occurred during periods of firing, particle concentration data was averaged for all of the instructors on the line at each base for both weapons and plotted against time (Fig. 5). Data represented are from M4/M9 classes at both bases and separated by weapon. Peaks in these plots are indicative of high particle concentrations while troughs indicate low particle concentration. When the peaks and troughs were evaluated by what the class was doing at the time using observational logs, peaks were associated with firing events while troughs were associated with gaps in firing (Fig. 5). This trend was apparent regardless of weapon type, indicating that firing events contribute more to instructor particle exposure (Fig. 5).

### Cu and LDSA were positively correlated with CO levels

**Fig. 6:**
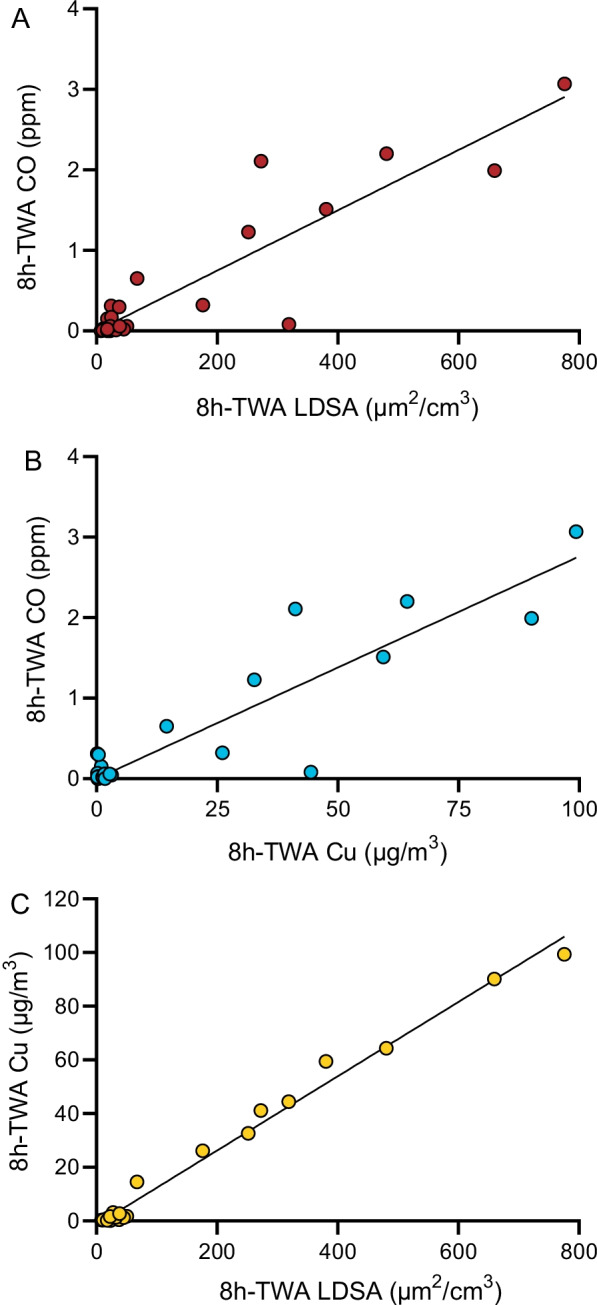
Positive correlations between 8 h TWA CO, Cu, and LDSA. **A** Correlation between 8 h TWA CO and LDSA, R^2^ = 0.836, *p* < 0.0001. **B** Correlation between 8 h TWA CO and Cu, R^2^ = 0.834, *p* < 0.0001. **C** Correlation between 8 h TWA Cu and LDSA, R^2^ = 0.989, *p* < 0.0001

Because both CO and LDSA measurements followed the same trends, we investigated whether there was a relationship between CO and LDSA. To do this, CO 8 h TWA values were plotted against LDSA 8 h TWA values for each individual measurement at both bases. This analysis yielded a significant positive correlation between the two variables (Fig. 6A). Additionally, when the 8 h TWA for CO was compared to the 8 h TWA for Cu, a significant positive correlation was also observed (Fig. 6B). Finally, there was a significant positive correlation when 8 h TWA for Cu was plotted against the 8 h TWA for LDSA (Fig. 6C).

### Lung function was maintained despite exposure

**Fig. 7:**
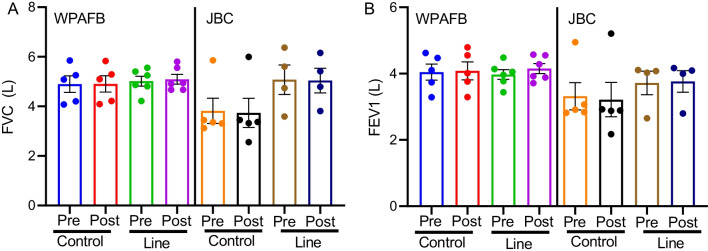
Lung function measured by spirometry. Spirometry tests were administered pre- and post- shift for both control participants and instructors on the line at both WPAFB and JBC. **A** FVC, **B** FEV1. Data are represented as individual points with the bars representing mean ± SEM

According to the Centers for Disease Control and the NIOSH, a normal, healthy adult will have a forced vital capacity (FVC) of approximately 3.8 L (female) and 5.3 L (male) and a forced expiratory volume after 1 s (FEV1) level of 3.3 L (female) and 4.3 L (male). In this study, there was no decrease in any lung function measurements in the instructor population post shift compared to pre shift for either FVC (Fig. 7A) or FEV1 (Fig. 7B). Additionally, there was no difference between the instructor group and the control group. Furthermore, all lung function parameters fell within the normal range.

### Oxidative DNA damage, 8-OGdG, was increased in instructors compared to controls

**Fig. 8:**
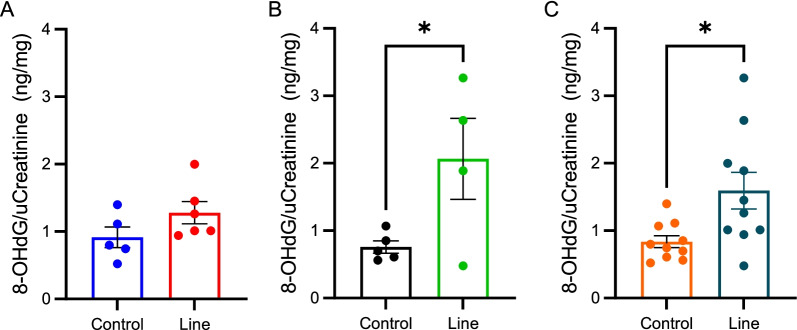
Urinary 8-OHdG levels. **A** WPAFB; **B** JBC; **C** WPAFB and JBC combined. *p < 0.05 v control. Data are represented as individual points with the bars representing mean ± SEM

8-OHdG is a common biomarker used to assess oxidative stress. Urine samples were collected before and after the work shift and post-shift 8-OHdG measurements were normalized to urinary creatinine (uCr) and then to each individual’s baseline allowing for the comparison between individuals and bases. At WPAFB, there was a trend for increased 8-OHdG levels in instructors on the line compared to controls (Fig. 8A, 1.24 ng/mg uCr vs 0.91 ng/mg uCr, *p* = 0.22). On the other hand, at JBC where exposure was higher, the 8-OHdG levels in line instructors compared to controls was significantly higher (Fig. 8B, 2.07 ng/mg uCr vs 0.76 ng/mg uCr, *p* = 0.045). Finally, when the data from both bases was analyzed together, line instructors displayed significantly higher 8-OHdG levels compared to controls (Fig. 8C 1.57 ng/mg uCr vs 0.84 ng/mg uCr, *p* = 0.022). Furthermore, when controlled for age, BMI, and gender, no significant associations to 8-OHdG outcomes were identified (Additional file 6: Table S3).

### Cu was increased in urine collected from instructors compared to controls

**Fig. 9:**
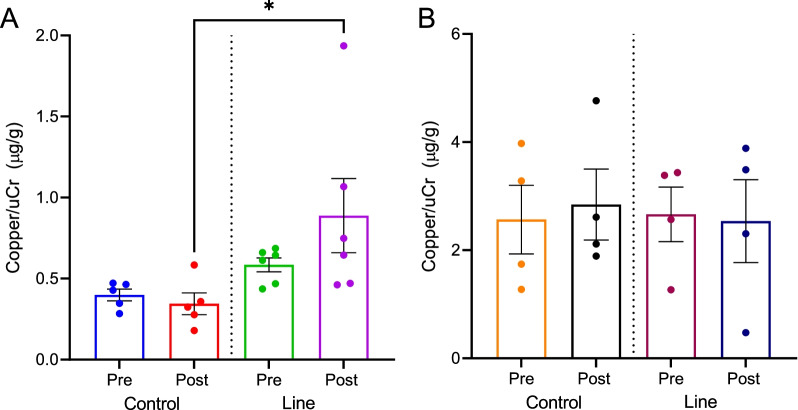
Urinary metal analysis. **A** WPAFB; **B** JBC. *p < 0.05 versus control post. Cu was normalized to urinary creatinine

In order to determine whether the evaluation of metals could be a viable biomarker of exposure, urine was collected both before (pre) and after (post) the work shift from controls and instructors and analyzed for metals. When specifically looking at Cu, instructors on the line at WPAFB had significantly higher Cu levels after exposure compared to controls (0.88 µg/g uCr vs 0.34 µg/g uCr, *p* = 0.046) (Fig. 9A). At JBC, no changes in Cu levels were detected in the urine from instructors after exposure compared to before exposure, although Cu levels, overall, were higher in all groups at JBC compared to WPAFB (Fig. 9B). When urinary Cu levels were adjusted for age, BMI, and gender, no significant associations were observed at either WPAFB or JBC (Additional file 7: Table S4). However, when a combined covariate analysis was performed, there was a significant effect of gender (*p* = 0.007) and a nearly significant effect of base, i.e. WPAFB *versus* JBC (*p* = 0.054) on urinary copper outcomes (Additional file 7: Table S4).

**Fig. 10:**
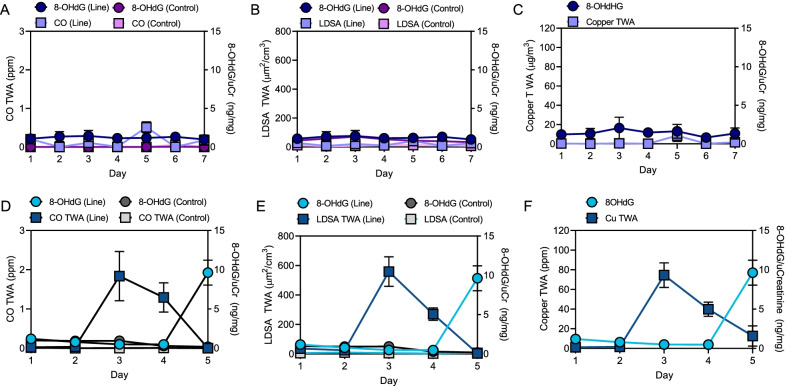
Simultaneous plots of air monitoring data and 8-OHdG levels over time. **A** 8 h TWA CO and 8-OHdG levels for line instructors and controls at WPAFB. **B** 8 h TWA LDSA and 8-OHdG at WPAFB. **C** 8 h TWA Cu and 8-OHdG at WPAFB. **D** CO and 8-OHdG levels for line instructors at JBC. **E** LDSA and 8-OHdG levels for line instructors at WPAFB. **F** LDSA and 8-OHdG levels for line instructors on the line at JBC

We also compared biological data to air monitoring data. Interestingly, when average daily 8-OHdG levels were plotted along with average daily CO 8 h TWA (Fig. 10A, D) and average daily LDSA 8 h TWA (Fig. 10B, E), it was clear that, at least at JBC, both CO 8 h TWA and LDSA 8 h TWA (dark blue lines) peaked at least a day and a half before 8-OHdG levels (light blue lines) peaked, (Fig. 10D, E). These results coincide with the M9/M4 night fire (day 3) and the make-up M4 class (day 4), again suggesting that exposure to M4 firing emissions results in a stronger biological response than exposure to M9 firing emissions. This trend was not observed at WPAFB (Fig. 10A, B). However, because the plots are represented with the same axis scales, it is easy to appreciate that the exposure at WPAFB was much less than observed at JBC. In fact, the exposure at WPAFB, at least for LDSA, seems to be on par with the control levels seen at JBC (Fig. 10B v E). The same trend was observed at JBC with Cu exposure, although, Cu was not detectable in the control samples which is why only line instructor data is represented (Fig. 10F).

## Discussion

Since the introduction of LFF ammunition in early 2000, USAF small arms firing range instructors have reported adverse symptoms including respiratory irritation, metallic taste, and sore throat despite exposure assessments concluding OELs are not typically exceeded [1, 4]. The primary objectives of this study were to generate a comprehensive characterization of LFF firing emissions at two different firing range types, to understand how exposure to these emissions affects range instructor health, and to identify biomarkers of exposure. Our approach was to measure the complex mixture of the weapons emissions using a variety of instruments and techniques. To do this, range ventilation was measured, symptoms were tracked, personal breathing zone areas were monitored for chemical exposures, and biological markers of exposure were measured to generate a complete exposure picture using both standard and state-of-the art technologies.

Proper firing range airflow is laminar and moves from the firing line down-range towards the bullet trap, where it is exhausted, moving firing emissions away from instructors and shooters. Improper ventilation design and function is a major contributor to exposure levels within firing ranges [1, 9]. WPAFB, a fully enclosed range, uses a rear-wall radial plenum to deliver airflow down-range, while JBC, a partially enclosed range, uses three rows of suspended fans (Fig. 1). At both ranges, air velocities were frequently outside the standard range of 0.381 ± 0.0762 m/s (75 ± 15 fpm) when measured using a nine-point grid in each firing stall and areas of stagnant and backflow were observed (Fig. 2). This is consistent with previous work at WPAFB where stagnant and backwards flow were observed despite average airflow velocities in the normal range [9].

Turbulent and reversed airflow likely contributes to exposure by moving the contaminants generated at the firing line back towards the instructors. Reverse airflow occurred most often at the edges of the range at both bases, and in front of the tower at JBC. At JBC, backflow was more pronounced on the right side of the range because two fans in the ventilation system behind lanes 17–20 were not operational during the sampling event (Fig. 2D and Additional file 2: Fig. S2). At WPAFB, backwards flow mainly occurred at the end of range in the first and last stalls (Fig. 2B and Additional file 1: Fig. S1). Smoke tests corroborated the issue at JBC, showing highly turbulent flow and backflow in front of the tower where there were no fans available to push air downrange (Fig. 2D and Additional file 2: Fig. S2).

Flow velocities were relatively consistent across measurement days at WPAFB but not at JBC. This can be explained by variation in weather and wind conditions at JBC due to the partially enclosed range design. On the right side of the range, more wind variability was observed which may have been influenced by the fact that two ventilation fans in this area were not working during the sampling period. Overall, wind speed was reduced compared to outdoor wind speeds suggesting that wind effect may be reduced in the partially enclosed range design, although not eliminated completely. The M4/M9 combined class was taught at night which may have contributed to wind changes compared to the M4 day class. It was also observed during M9/M4 firing that the wind direction caused firing emissions to move laterally across the firing line rather than down-range, increasing exposure to instructors. Shifts in wind direction and magnitude could result in air being drawn out of the range towards the firing line or pulled laterally across the range thereby increasing exposure. Overall, neither WPAFB nor JBC had adequate ventilation to produce the desired flow velocities; however, the increased backflow at JBC may have been influenced by outdoor weather conditions in addition to several ventilation fans being inoperable at the time of sampling.

Prior studies have found that metals, primarily Cu and zinc, the major components of the projectile and jacket, are a significant component of firing emission byproducts [6, 12]. To more closely investigate the exposure of instructors to various metals, particles in the breathing zone were collected on filters and analyzed for metals. Cu was by far the most prevalent metal detected after all classes at both bases (Fig. 3 and Additional file 5: Table S2). Furthermore, the respirable Cu measurement was approximately equivalent to total Cu, indicating that the majority of Cu particles were found to be in the respirable fraction (Fig. 3C, E), which is consistent with previous work on LFF firing emissions [1, 12]. At JBC, Cu exposures were up to tenfold higher than at WPAFB (Fig. 3A). Although total Cu emissions did not reach the OSHA 8 h TWA PEL of 0.1 mg/m^3^, they did pass the action limit, the point at which steps must be taken to monitor the exposure levels of workers, of 0.05 mg/m^3^ during M9/M4 firing at JBC (Fig. 3A). Overall, Cu exposure at both bases was higher during the combined M9/M4 classes (Fig. 3A). This could be due to the fact that more rounds of ammunition were shot during the combined class.

CO levels, on average, were significantly higher at JBC compared to WPAFB for both peak (4 times higher) and 8 h TWA (6.5 times higher) (Fig. 4A, B). Consistent with metal and particle data, CO exposure was also highest during the M4/M9 class, although this difference was significant at JBC compared to all WPAFB classes (Fig. 4A). In fact, peak CO levels for several instructors at JBC either approached or surpassed the ACGIH ceiling limit of 125 ppm. During the M4 class at JBC, one instructor experienced a peak CO exposure level of 142 ppm (Fig. 4B). Additionally, during M4/M9 firing at JBC, three of the four instructors observed peak CO levels of 103 ppm, 110 ppm, and 111 ppm. Interestingly, one instructor had a peak CO reading of only 15 ppm during the combined class. This individual was instructing on the left-most side of the range during that class suggesting that, in this instance, weather may have contributed to the wide variability observed at JBC. This is further supported by the fact that the instructor on the right side of the range had the highest peak CO level observed that night (111 ppm). It is important to note that there was no physical barrier between the tower and the firing line at JBC (Fig. 1B) resulting in the tower instructor also being exposed. In fact, during the M4/M9 class at JBC, the tower instructor had the second highest peak CO observation (110 ppm) demonstrating that the exposure to the tower was exacerbated by the negative airflow detected in front of the tower.

Particle concentration was distributed in sharp peaks that corresponded to firing events (Fig. 5) with the highest peak particle exposures observed during M9/M4 combined classes at both bases (Fig. 4C, D). At JBC, peak M9/M4 particle concentrations were approximately 1.5 times higher than M4 and 2.5 times higher than M9. Furthermore, both peak and 8 h TWA particle concentrations at JBC were significantly higher for all class types compared to WPAFB. UFPs were also evaluated in real-time using a personal electrometer and, during sampling, showed large increases to total particle concentration during firing events (Fig. 5). This observation, consistent with prior literature [12], is particularly important because UFPs are known to deposit in the gas exchange area of the lungs where they can cause respiratory disease [15, 16]. Additionally, due to their small size, UFPs are able to translocate into the bloodstream where they can travel to other organs causing damage [15, 18, 19]. Together, these data highlight the notion that current OELs for Cu and CO are not sufficient to assess occupational exposure of emissions to range instructors as symptoms still develop (Table 4) despite not reaching the OEL. Additionally, standards for UFPs are needed given that substantial exposure comes from these particles. On the other hand, we detected a positive correlation between the 8 h TWA of CO and LDSA and CO and Cu (Fig. 6) suggesting that perhaps CO may be a surrogate measure for UFPs and/or Cu that could be implemented.

Real-time CO concentrations may prove useful in assessing the effectiveness of the ventilation system in controlling airborne contaminants generated during weapons firing indoors, particularly in partially enclosed ranges. Furthermore, this study demonstrates that firing emissions generated from M4 rifle firing were higher compared to the M9 pistol. This trend was observed when analyzing Cu, CO, and UFPs and was consistent across both firing ranges. Together, these data demonstrate that exposure generated from the M4 rifle is worse than the M9 pistol.

Daily symptom questionnaires supported previous evidence that despite OELs rarely being exceeded, range instructors still experienced adverse effects from exposure to LFF firing emissions (Table 4). Moreover, symptom reports collected from all instructors on the line per class appeared to increase with hours of exposure and rounds fired per student. Finally, the clustering of symptoms around a single individual at each base suggests that some individuals may be more sensitive to emissions from LFF ammunition. Additional research that would allow for increased sample sizes and length of observation per base may further clarify these trends and yield useful information.

To understand the health effects of firing emission exposures, urine was collected from instructors and controls before and after each work shift. It is well known that UFP exposure causes an increase in oxidative stress and a mild but detectible decline in lung function [18, 29, 30]. However, in the current study no such decline in lung function was detected (Fig. 7). This is consistent with a study by Voie et al. where no appreciable decline in lung function was observed at CO and dust concentrations of 241 ppm and 17.3 mg/m^3^ respectively [6]. Together, these results suggest that monitoring lung function is not sufficient to detect health effects of LFF ammunition in instructors given that instructors reported respiratory symptoms.

On the other hand, urinary oxidative stress measurements (8-OHdG) showed an increase among instructors compared to controls and this was significant at JBC, where CO and LDSA exposures were 5.8 and 11.2 times higher respectively than at WPAFB, and when instructors and controls from both bases were combined (Fig. 8). Oxidative DNA damage is known to be associated with the development of many diseases including cancer, cardiovascular disease, and respiratory diseases [31]. Taken together, it is clear that instructors on the line at both bases have increased oxidative stress compared to controls. While this increase cannot be directly linked to UFP exposure, these results do show that there is a stress response occurring within the body following firing emission exposure that is absent in the control population. Furthermore, urinary 8-OHdG levels may be a viable non-invasive means to monitor instructor reaction to firing emissions over time.

Cu was also detectable at significantly increased levels in urine collected from instructors at WPAFB post-shift compared to controls (Fig. 9A). However, at JBC, there was no change in urinary Cu between all of the groups (Fig. 9B). This may be because the temperatures at JBC were consistently in the mid to high 80 s during the study period leading to the instructors hydrating and urinating more frequently. Furthermore, gender was significantly associated with urinary Cu outcomes when the data from both WPAFB and JBC was combined together (Additional file 7: Table S4). This is likely due to the low population sizes and mismatched gender ratios, which is a limitation of this study. Cu is known to be a respiratory irritant, which is why exposure standards exist; however, in the current study, respiratory symptoms were reported despite Cu levels not reaching the OEL. Several studies have shown that Cu and copper oxide (CuO) particles in the ultrafine size range can cause increased cell death and increased oxidative stress [32, 33]. Additionally, CuO UFPs were more readily taken up by lung cells than CuO microparticles leading to increased DNA damage and cell toxicity [33, 34]. Because Cu particles detected in this study are in the ultrafine, respirable range, new standards need to be developed to address UFP exposure, which may be a primary contributor to instructor symptoms.

Finally, the relationships between biological markers and personal exposure measurements were assessed. Interestingly, exposures occurred at JBC about 1.5 to 2 days prior to the 8-OHdG peak (Fig. 10). In a study where rat mesothelial cell lines were treated with varying concentrations of asbestos, 8-OHdG levels peaked 72 h after treatment compared to 24 and 48 h [35]. Furthermore, the higher the exposure, the more this trend became apparent [35]. It is possible that the higher exposure experienced at JBC results in better visualization of the delay in biological detection compared to WPAFB.

## Conclusion and future directions

The results of this research demonstrate that exposure to instructors from LFF firing emissions is complex and multifaceted. By far, the most significant recommendation that can be made to directly mitigate exposure is to monitor ventilation regularly to ensure that airflow is consistent across the firing line and within the standard range of 0.381 ± 0.0762 m/s (75 ± 15 fpm) and that backflow is eliminated. NIOSH recommends ventilation system airflow measurements every three months to increase the effectiveness of air contaminant control within firing ranges and provide timely notification of ventilation failures so that they can be repaired [4]. At WPAFB and JBC, the majority of the stalls had air velocity measurements that were outside the recommended range. At a minimum, stalls where backflow is observed should not be used for training to protect the health of range instructors. At JBC, it is recommended that ventilation fans be installed above the tower as this is an area of high backflow, increasing exposure to the tower instructor.

The results from this study show that the current mass-based Cu OELs are not an adequate metric to protect instructor health. Despite OELs not being exceeded, instructors still exhibited adverse symptoms along with measurable increases of metals and oxidative stress markers in urine. Routine monitoring of symptoms, urinary metals, and oxidative stress biomarkers can help identify instructors who are particularly affected by exposures. These results can assist in guiding protective strategies to reduce instructor exposure.

Specifically, it is recommended that personal exposure be measured on a regular basis. Because CO, metals (in particular, Cu), and UFPs are positively correlated with each other (Fig. 6), exposure levels to firing emissions may be approximated by monitoring one of these chemicals. Real-time personal monitoring of UFP exposure would be ideal, as it is clear from the literature that UFPs dominate firing emissions and present a significant health risk; however, there is currently no exposure standard for UFPs.

In the absence of an exposure standard, and due to the high cost of UFP monitors (~ $10 K), we recommend real-time personal monitoring of CO exposure as a marker for exposure to firing emissions. CO monitoring allows for real-time feedback regarding instructor locations, firing patterns, and weather/ventilation conditions leading to higher exposure levels. At a minimum, CO should be measured on days where the M4 rifle is fired as the data from this study show that the M4 firing produces more emission in the breathing zone of instructors than M9 firing alone.

Further, additional work is necessary to understand the long-term ramifications of exposure to LFF emissions. This study is limited by a small population size and short study duration. Longitudinal studies need to be completed to understand potential disease consequences of exposure. Additionally, more work needs to be completed to understand the health effects of concurrent exposures (i.e. Cu, CO, and UFPs). A competing factor in long term health effects not measured by this study was noise exposure. Although double hearing protection, including foam ear plugs and safety ear muffs, was worn by all subjects, evidence from other epidemiological studies suggest that frequent exposure to noise levels below the safety standards can contribute to cardiovascular health effects [36, 37]. Future studies focusing on comprehensive exposure assessment should incorporate measurements of noise as well as breathing air contaminants.

## Methods

### Sampling locations and weapons

Sampling was completed at a fully enclosed small arms firing range at WPAFB in Dayton, Ohio and a partially enclosed small arms range at JBC in Charleston, South Carolina. These bases were selected to assess exposure at small arms ranges with different types of ventilation systems. At the fully enclosed range at WPAFB, a perforated ventilation plenum at the top of the back wall provides airflow into, across the ready/firing line, and downrange towards the bullet trap. The plenum delivers air provided by two air handling systems, each providing airflow for one half of the range. All areas are located indoors, and the ventilation system is designed to keep the firing range at a slightly negative pressure. The tower where an instructor provides instruction to the class, is physically separated from the range, and climate control in this room is provided by a separate air handling system. Observation of the firing line is made through bullet-proof windows.

Unlike the small arms range at WPAFB, the partially enclosed range at JBC utilizes a ventilation system comprised of individual, tube-axial ventilation fans. The ventilation system consists of three banks of ten fans each, located just under the roof deck at the back, middle, and end of the range above the bullet trap. This orientation of fans is intended to move air from the firing line toward the bullet trap. The firing line in the partially enclosed range is open to the outdoors, and the roof above the bullet trap is composed of separated pieces, allowing emissions to exhaust out of the range. Also, unlike at WPAFB, the tower is not isolated from the range in a separate room, thus the tower instructor (located just behind the ready line) is also exposed to firing emissions.

At both bases, instructors stand between a yellow ready line, roughly six feet removed from the red firing line where shooters stand to fire their weapons during training classes. For this study, the range instructors attempted to maximize class numbers. Weapons included the M9 pistol, a single-round, semiautomatic weapon with a barrel length of 125 mm, and the M4 rifle, which can be operated in single-round or three-round burst mode and has a barrel length of 370 mm. M4 and M4/M9 combined training classes fired the rifle in both modes. Both weapons utilized LFF ammunition.

### Ventilation assessment

During the study at WPAFB, ventilation assessments were performed daily prior to firing classes. To account for the differences in ranges and ventilation system design, two different approaches were used to measure airflow velocity and direction. Nine-point measurements, in a grid-like fashion, were taken within each stall across the firing line and at the walls using three Shortridge Instruments Air Data Multimeters model 880-C using the Velgrid attachment. These instruments were used as opposed to standard hot wire anemometers because they indicate direction of flow in addition to velocity. The Velgrids average the air velocity across 16 points in a 30.48 × 30.48 cm square. The nine-point measurements consisted of three readings each on the left, middle, and right side of each stall at heights of 0.305 m, 0.914 m, and 1.524 m. In order to make the measurements time effective, a cart was constructed which allowed for simultaneous capture of readings at each height.

Using the same measurement technique, ventilation measurements were made at JBC prior to three of the four days of firing due to time constraints. Additionally, wind speed and direction were measured during the M9/M4 combined class and M4 class in order study the effect of wind on emissions. Four Kestrel 5500 Pocket Weather Trackers (Nielsen-Kellerman) were placed two meters behind the instructing line, measuring wind speed and direction with a frequency of 1 Hz. The Kestrels were placed behind lanes 1 (range left), 8 (tower left), 13 (tower right), and 20 (range right). Only three of the four weather trackers recorded data, and as such results are shown with the three locations recorded.

A Degrees Control Inc. C-Breeze artificial fog generator was used to qualitatively measure flow direction at both ranges by walking across the firing line at each stall [1, 9] (Additional file 1: Fig. S1 and Additional file 2: Fig. S2).

### Participant inclusion and data collection

Only active-duty AF members were eligible to participate in the study. Range instructors were recruited from Security Forces at both WPAFB (n = 9) and JBC (n = 4). However, due to reassignments, attendance, and underlying health issues, two instructors from WPAFB and one instructor from JBC were excluded. Additionally, control participants were recruited from Security Forces at each base (WPAFB, n = 5; JBC, n = 5). Across both bases, a total of 11 instructors and 10 controls were included during analysis of survey and biomarker data. All participants were assigned a unique identifier under which their data and samples were logged.

Range instructors at WPAFB were monitored during their workday from 08 Mar 2019 to 20 Mar 2019, and control participants were monitored from 29 Mar 2019 to 10 Apr 2019. The study at JBC took place on 08 Jul 2019 through 19 Jul 2019 with instructors being monitored the first week and controls the second week.

Monitoring included daily questionnaires and a pre-study initial questionnaire, real-time personal breathing zone measurements for metals, CO, and UFPs during the work shift and physiological lung function measurements (pulmonary function test) and biological sample collection (urine) before and after the work shift. This study was reviewed and approved by the Air Force Research Laboratory (ARFL) institutional review board (IRB) (FWR20190025N).

### Questionnaires

A questionnaire series was constructed on the Survey Monkey platform to capture symptom type and frequency along with lifestyle factors to support interpretation of environmental and biological data. The series consisted of an initial one-time background questionnaire, a daily pre-work questionnaire, and a daily post-work questionnaire. Content design was informed by prior studies of AF Combat Arms populations [1] along with literature on potential biomarker confounders related to lifestyle and health factors [38–42]. Occupational health, medical, and range experts reviewed the questionnaires before they were finalized. Additionally, phrasing was adjusted to align with each base’s operations and tempo.

All questionnaires were administered electronically via Apple iPad tablets connected to the Survey Monkey mobile application, and responses were logged under the participant’s unique identifier. On the first day of sampling, participants responded to the background questionnaire upon arriving at the workplace, lasting up to 25 min. It captured data related to demographics (age, race, sex, height, weight, rank); lifestyle factors (tobacco use, hobbies, off-duty ammunition use); health status (allergies, medications/supplements, hereditary conditions, dietary factors); occupational history (years in current role, years at base, past duties); past symptom experiences; and current state (exposures within 24 h, active illness or symptoms). Recall timeframes for behavior and health history questions were mostly within the past week while symptomology was within the last month. Due to time constraints, collection of these data topics on the first day at JBC was split between the initial pre- and post-shift questionnaires.

Subsequent pre-shift and post-shift questionnaires were shorter, lasting up to 5 min. These questionnaires were administered daily before and after work shifts, often in conjunction with biological sample collection. Content and administration of all questionnaires for control populations mirrored that of instructors, minus the collection of firing range details. Pre-shift questionnaires captured off-duty behaviors since the prior shift, recent consumption of products associated with inflammatory markers (caffeine, tobacco, medication, etc.), symptoms experienced since prior shift, and range assignment (instructors only). Post-shift questionnaires captured duty details from that day and any symptoms experienced during shift. Responses were exported from Survey Monkey to Excel at the end of the sampling period for data management and visualization. Each participant’s responses were linked to environmental and biological sample data by the individual’s unique identifying code for analysis.

### Personal exposure monitoring

Each participant (instructor and control) was issued a personal exposure vest (PEV) prior to beginning their workday and the vest was collected at the end of each workday. PEVs included four pieces of equipment to characterize exposure, including two air sampling pumps connected to filter cassettes to collect particulates for offline analysis of metals, a direct reading gas meter, and a direct reading UFP sampler.

Particulates were collected and analyzed offline for metals using NIOSH 7303. Briefly, two 37 mm MCE filter cassettes were attached to the lapel on each vest and connected to the air sampling pump via Tygon® tubing. One sample was collected for total metals and a second cassette was outfitted with an aluminum cyclone filter (Zefon International) to capture respirable particulate (cut size of 4 µm). Air was pulled through the MCE cassettes via GilAir (Sensidyne) personal sampling pumps with flowrates at 2.5 L per minute. Pumps were calibrated using a Defender (DryCal®) primary flow calibrator within 5% of desired flowrates before and after each sampling event. If post-calibration of pumps exceeded a 5% difference from pre-calibration, sample volumes were adjusted to reflect the new calibration. The filters were analyzed for metals by ALS Environmental using inductively coupled plasma mass spectrometry (ICP-MS).

CO was monitored by a MultiRAE Pro (RAE Technologies) and data was logged every second for the duration of the exposure. UFPs were monitored using the Partector® (Naneos). This device computes the exposure metric known as the lung deposited surface area (LDSA), which is a measure of the concentration of an aerosol that will deposit in the lung based on the size of the particles [43]. The Partector measures LDSA from 0 to 12,000 µm^2^/cm^3^ with an average particle diameter < 300 nm. It also estimates particle number concentration from 0 to 1 × 10^6^ particles per cm^3^ of air.

During firing events, instructor locations were noted to help understand how personal exposure data related to ventilation assessment data. The control population was sampled in the same manner as instructors, though specific location notes were not taken by observers. Location logs were given to the control subjects, so that they could identify their location over the course of the day to explain any anomalies in exposure data.

### Pulmonary function testing

Individual forced vital capacity (FVC) and first second expiratory volume (FEV1) were measured by spirometry using the Spirodoc (Medical International Research) according to manufacturer instructions. Pulmonary measurements were made pre-shift and post-shift. Post-shift measurements were compared to pre-shift measurements and analyzed for significant differences.

### Urine collection and analysis

Urine was collected pre-shift and post-shift from both instructors and controls every workday. Upon arrival to the collection area, participants were provided with a urine specimen cup pre-labeled with their unique identifier. Participants provided a clean catch urine sample which was immediately placed into a secondary Ziploc® bag and stored on ice until transport to the lab. At the lab, a 5 mL aliquot of each bulk urine sample was transferred to a 15 mL conical tube that was prelabeled with the participant unique identifier, date and pre- or post- collection time. Urine samples were centrifuged at 2000 × g for 15 min at 4 °C and the resulting supernatant was aliquoted into 1 mL volumes for analysis or stored at -80 °C.

Levels of urinary 8-OHdG were measured by a commercial enzyme linked immunosorbent assay (ELISA) kit (R&D Systems) according to manufacturer instructions. Briefly, urinary supernatant was diluted 1:10 and 25 µL of the diluted sample was loaded per well in duplicate in a 96-well plate. A standard curve was run on each plate. To calculate the concentration of 8-OHdG, the standard curve values were log transformed, plotted, and fit with a polynomial regression line. 8-OHdG levels were normalized to uCr, which was measured using both commercial (Arbor Assays, R&D Systems) and in-house colorimetric assays. The same urine samples used for 8-OHdG analysis were diluted 1:20 in distilled/deionized water and 50 µL of sample was run per well in duplicate. Similar to the 8-OHdG assay, a standard curve was run on each plate. An alkaline picric acid solution was generated by combining 2.5 mL of 1 N NaOH with 12.5 mL of 0.13% Picric Acid and 100 µL was added to each well. After 45 min, the absorbance was read at 490 nm. The standard curve was log transformed and fitted with a linear regression line in order to calculate uCr levels. 8-OHdG levels were divided by the uCr levels for normalization. All individual post-shift 8-OHdG/uCr levels were then normalized to the initial pre-shift level (Monday pre) as a true baseline. Urine specimens that were not initially spun down were sent to the University of Cincinnati for metallomics analysis by ICP-MS. Metallomic results were normalized to uCr levels.

All biological samples collected at WPAFB were processed and stored after each shift. While at JBC, samples were stored at -20 °C until specimens were shipped back to the lab for processing.

### Statistical analysis

Significance of demographic and lifestyle differences between bases and participant groups were analyzed using T-tests for continuous variables and using Fisher’s Exact tests for categorical variables due to small sample sizes. Pearson’s correlation calculations were used to assess relationships between symptom reports, range class characteristics, and instructor characteristics. An alpha of 0.05 was used to determine statistical significance. All survey data was analyzed using SAS software, Version 9.04.

Personal exposure monitoring results were reported as the mean ± the standard error of the mean (SEM). T-tests were used for statistical significance analysis between two groups. T-tests assuming unequal variance were used on data from real-time measurements attached to PEVs, significance was obtained when *p* < 0.05 unless indicated otherwise. For comparisons between multiple groups, a one-way analysis of variance (ANOVA) with Tukey’s post-hoc test was used.

Biological results were reported as the mean ± SEM. For comparisons among multiple groups, ANOVA with Tukey’s post-hoc test was used. For comparisons between groups, Student’s t-test was used for significance analysis. Pearson’s correlation calculations were used to identify correlations between measurements. Statistical significance was obtained when *p* < 0.05 unless indicated otherwise.

Multiple linear regression analyses were performed to adjust age, gender, and BMI effect on urinary Cu and 8-OHdG levels. The difference between pre and post exposure levels of Cu or 8-OHdG were used as the outcomes in the linear regression analyses. Cu was normalized and measured daily, while 8-OHdG was normalized and measured relative to a pre-exposure baseline date. We performed strata level and combined analyses that included age, gender, and BMI as covariates. Additionally, a combined analysis was performed where the Base (WPAFB or JBC) was used as a covariate. These analyses were performed using R version 4.0.4 in RStudio and a *p* value < 0.05 was considered statistically significant.

## Supplementary Information

**Additional file 1: Fig. S1**. Representative images of qualitative fog ventilation measurements at WPAFB. Representative images of the airflow in each stall of the fully enclosed range through use of a fog generator. Numbers on images represent firing stall numbers.

**Additional file 2: Fig. S2**. Representative images of qualitative fog ventilation measurements at JBC. Representative images of the airflow in each stall of the partially enclosed range through use of a fog generator. Numbers on images represent firing stall numbers. Lanes 10-13 are located in front of the tower.

**Additional file 3: Fig. S3**. Wind speed and direction during two classes at JBC. **A**. Wind speed and direction frequency during the M4/M9 combined class. **B**. Wind speed and direction frequency during the M4 class. Direction is indicated as where the wind is coming from pointing to the direction the wind is blowing. The frequency is indicated by the length of the shaded sections which correspond to wind speed.

**Additional file 4: Table S1**. Primary Duties of Control Population by Base.

**Additional file 5: Table S2**. Average Metal Exposure to Security Forces (all values in µg/m³).

**Additional file 6: Table S3**. Multiple linear regression model for urinary 8-OHdG level estimations.

**Additional file 7: Table S4**. Multiple linear regression model for urinary Cu level estimations.

## Data Availability

The datasets used and/or analyzed during the current study are available from the corresponding author on reasonable request.

## Abbreviations

ACGIH: American Conferences of Governmental Industrial Hygienists
AF: Air Force
AFRL: Air Force Research Laboratory
ANOVA: Analysis of Variance
BMI: Body Mass Index
CO: Carbon monoxide
CO_2_: Carbon dioxide
Cu: Copper
CuO: Copper oxide
DoD: Department of Defense
ELISA: Enzyme-linked immunosorbent assay
FEV1: First second Expiratory Volume
fpm: Feet per minute
FVC: Forced Vital Capacity
HCl: Hydrogen chloride
HCN: Hydrogen cyanide
HPW: Human Performance Wing
ICP-MS: Inductively Coupled Plasma Mass Spectrometry
IRB: Institutional Review Board
JBC: Joint Base Charleston
LDSA: Lung Deposited Surface Area
LFF: Lead-Free Frangible
MCE: Mixed Cellulose Ester
n: Number of samples
NH_3_: Ammonia
NIOSH: National Institute of Occupational Safety and Health
NO: Nitrogen monoxide
NO_2_: Nitrogen dioxide
OEL: Occupational Exposure Limit
OSHA: Occupational Safety and Health Administration
PEL: Permissible Exposure Limit
PEV: Personal Exposure Vest
ppb: Parts per billion
ppm: Parts per million
r: Correlation Coefficient
R^2^: Coefficient of Determination
Redox: Reduction–Oxidation Reaction
ROS: Reactive Oxygen Species
SD: Standard Deviation
SEM: Standard Error of the Mean
SFS: Security Forces Squadron
TLV: Threshold Limit Value
TWA: Time Weighted Average
uCr: Urinary Creatinine
UFP: Ultrafine Particle
USAF: United States Air Force
VOC: Volatile Organic Compound
WPAFB: Wright Patterson Air Force Base
wt: Weight
8-OHdG: 8-Hydroxy deoxyguanosine
