# Supplementary figures and images for: Exposure to lead-free frangible firing emissions containing copper and ultrafine particulates leads to increased oxidative stress in firing range instructors
Source: Part Fibre Toxicol. 2022 May 15;19:36. doi: 10.1186/s12989-022-00471-0 (PMC9107651; doi:10.1186/s12989-022-00471-0)

## Slide 1
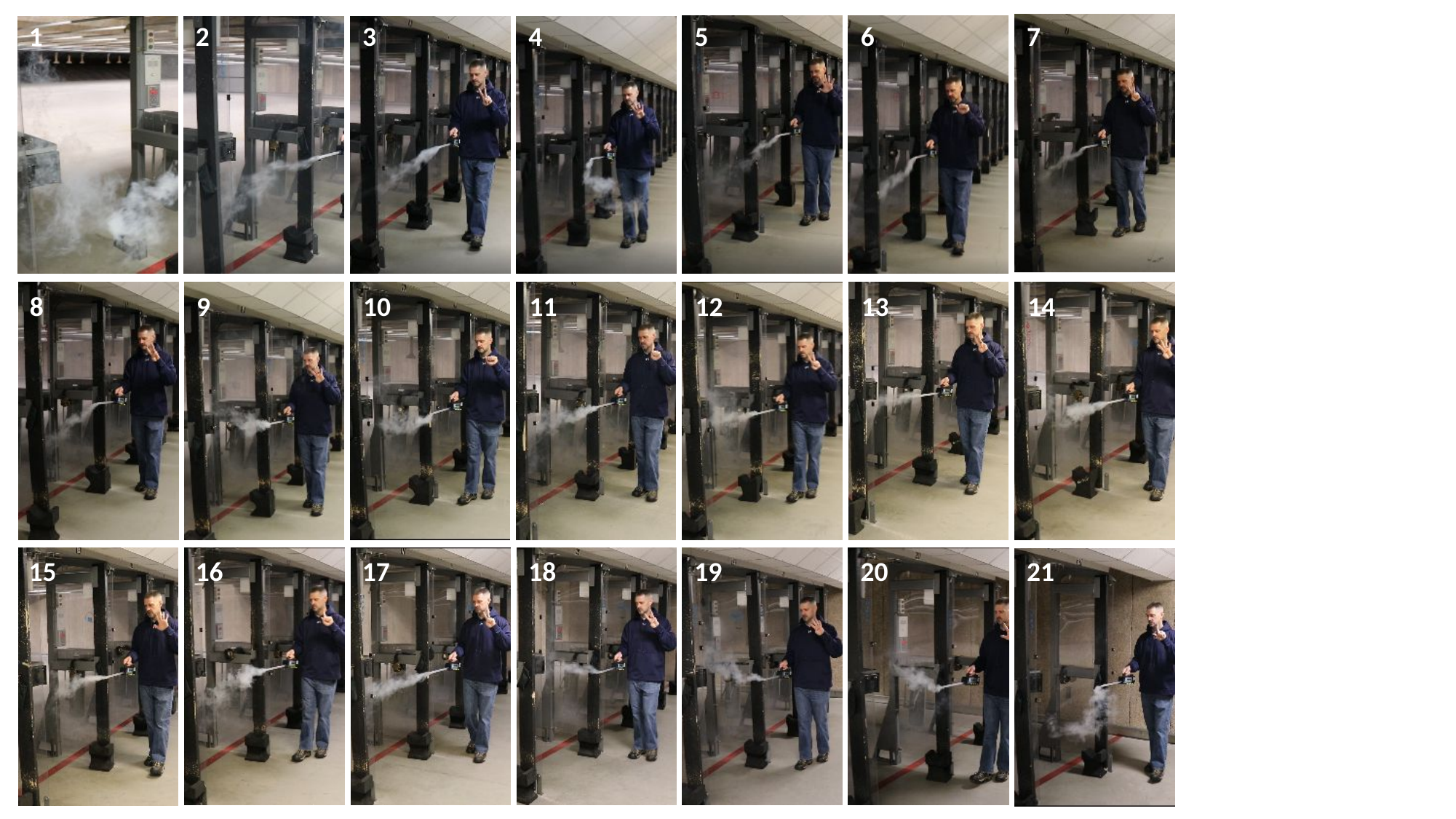

1
2
3
4
5
6
7
10
11
12
13
14
8
9
15
16
17
18
19
20
21

Supplement: Supplementary file 1 — Additional file 1: Fig. S1. Representative images of qualitative fog ventilation measurements at WPAFB. Representative images of the airflow in each stall of the fully enclosed range through use of a fog generator. Numbers on images represent firing stall numbers. [file 12989_2022_471_MOESM1_ESM.pptx]

## Slide 1
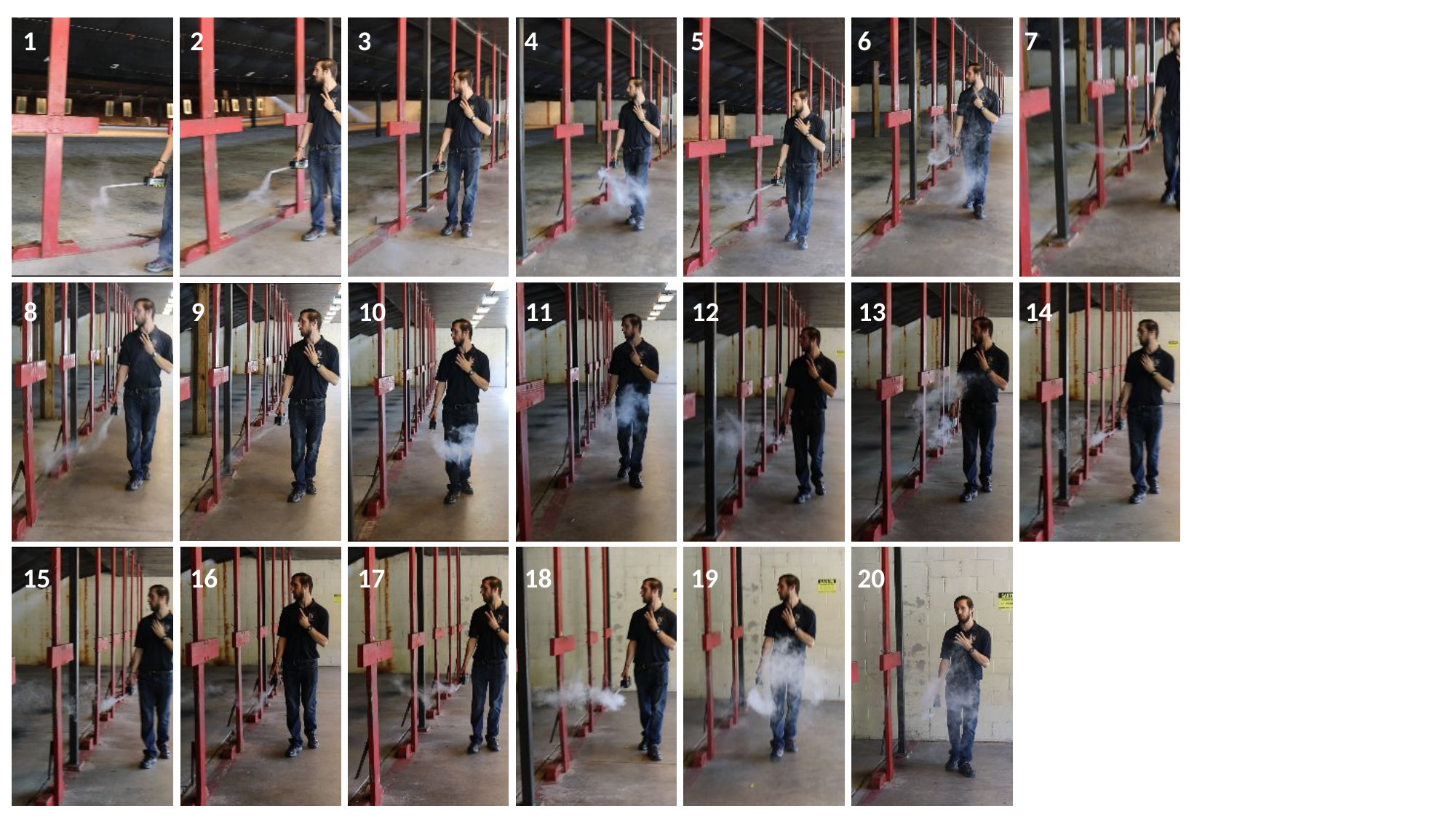

1
2
3
4
5
6
7
10
8
9
11
12
13
14
15
16
17
18
19
20

Supplement: Supplementary file 2 — Additional file 2: Fig. S2. Representative images of qualitative fog ventilation measurements at JBC. Representative images of the airflow in each stall of the partially enclosed range through use of a fog generator. Numbers on images represent firing stall numbers. Lanes 10-13 are located in front of the tower. [file 12989_2022_471_MOESM2_ESM.pptx]

## Slide 1
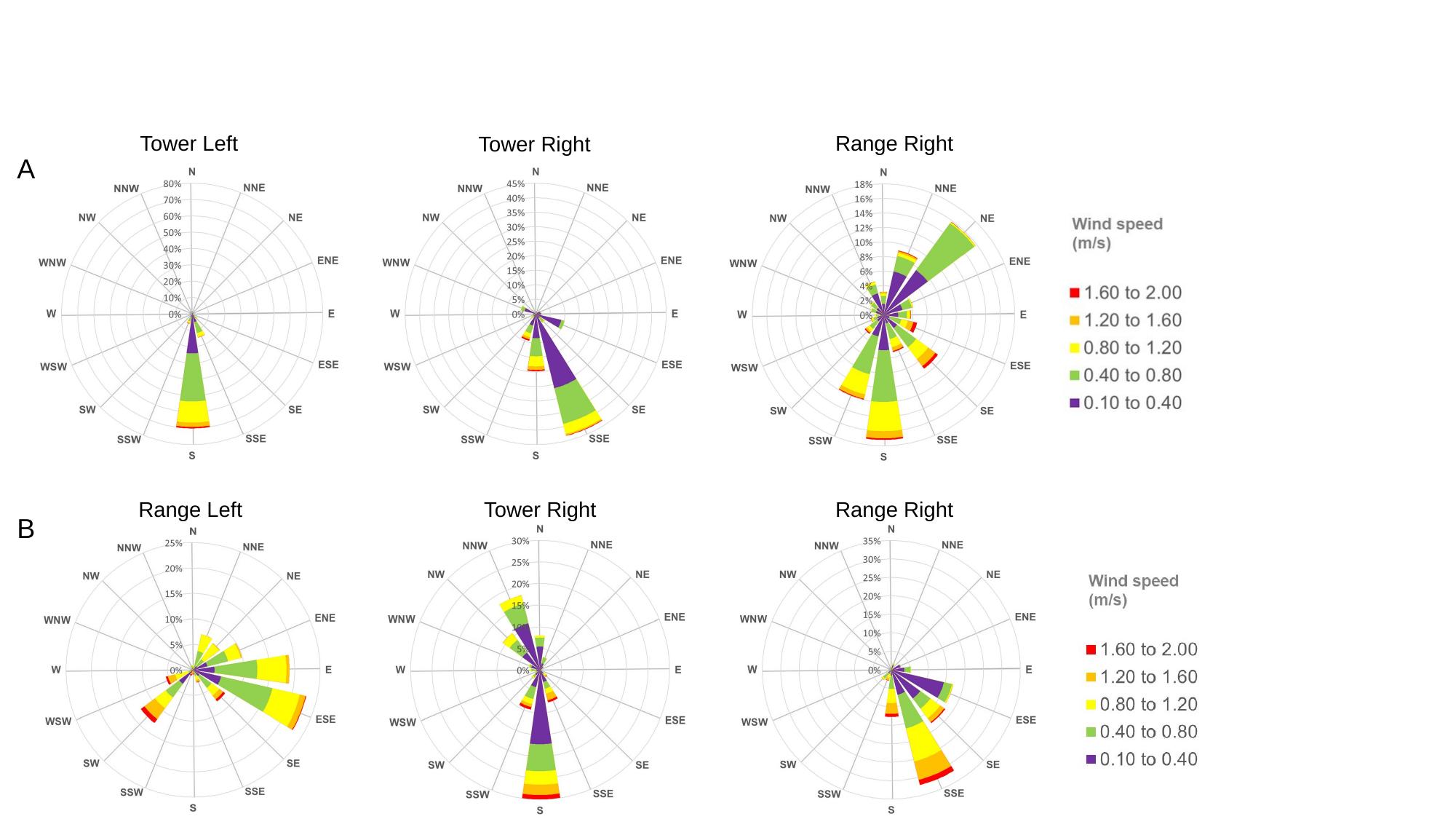

Tower Left
Range Right
Tower Right
A
Range Left
Tower Right
Range Right
B

Supplement: Supplementary file 3 — Additional file 3: Fig. S3. Wind speed and direction during two classes at JBC. A. Wind speed and direction frequency during the M4/M9 combined class. B. Wind speed and direction frequency during the M4 class. Direction is indicated as where the wind is coming from pointing to the direction the wind is blowing. The frequency is indicated by the length of the shaded sections which correspond to wind speed. [file 12989_2022_471_MOESM3_ESM.pptx]
