# Supplementary material for: Exposure to lead-free frangible firing emissions containing copper and ultrafine particulates leads to increased oxidative stress in firing range instructors
Source: Part Fibre Toxicol. 2022 May 15;19:36. doi: 10.1186/s12989-022-00471-0 (PMC9107651; doi:10.1186/s12989-022-00471-0)
Supplement: Supplementary file 4 — Additional file 4: Table S1. Primary Duties of Control Population by Base. [file 12989_2022_471_MOESM4_ESM.pptx]

## Slide 1
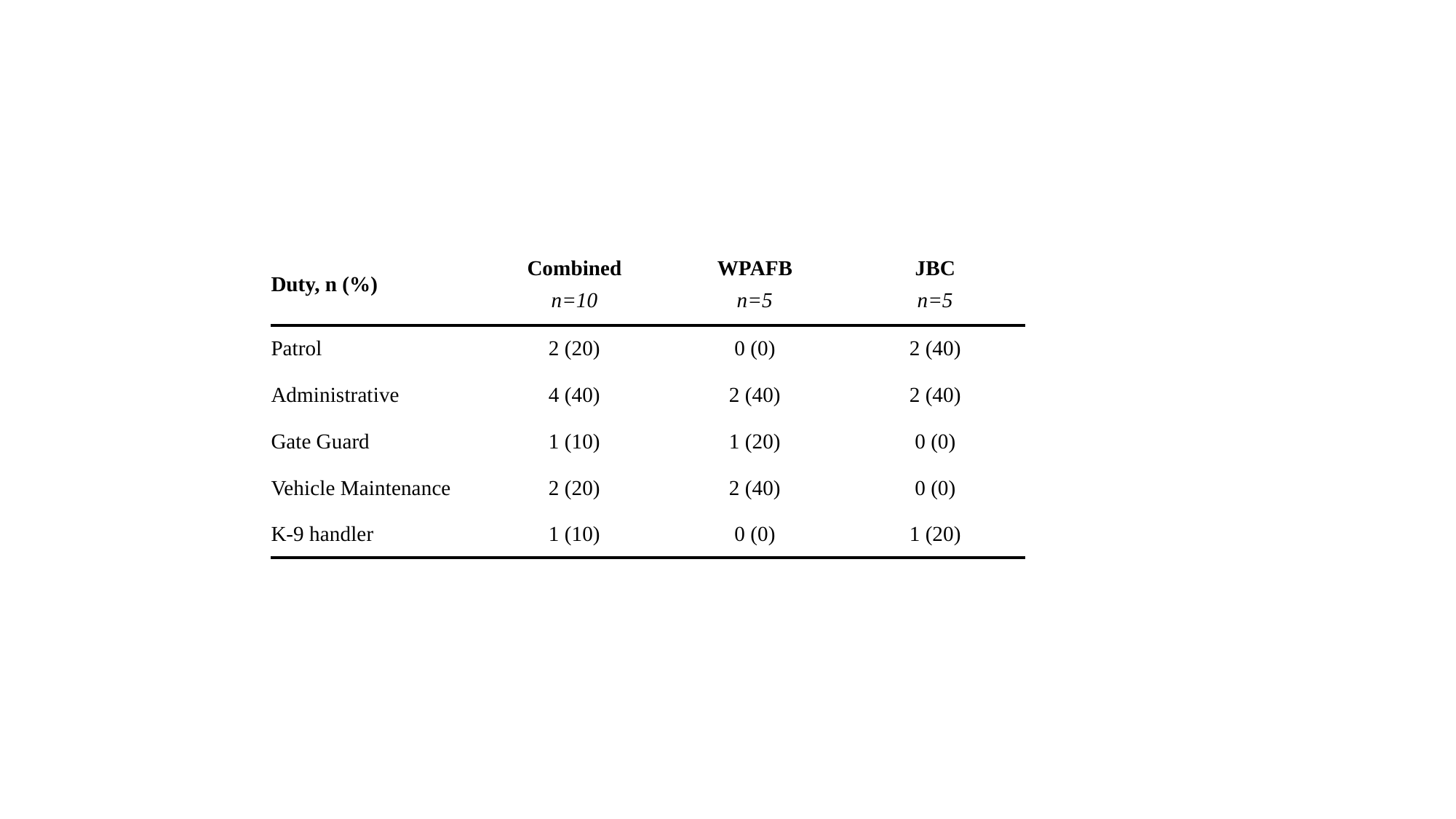

| Duty, n (%) | Combined n=10 | WPAFB n=5 | JBC n=5 |
| --- | --- | --- | --- |
| Patrol | 2 (20) | 0 (0) | 2 (40) |
| Administrative | 4 (40) | 2 (40) | 2 (40) |
| Gate Guard | 1 (10) | 1 (20) | 0 (0) |
| Vehicle Maintenance | 2 (20) | 2 (40) | 0 (0) |
| K-9 handler | 1 (10) | 0 (0) | 1 (20) |
| | | | |
