# Supplementary material for: Exposure to lead-free frangible firing emissions containing copper and ultrafine particulates leads to increased oxidative stress in firing range instructors
Source: Part Fibre Toxicol. 2022 May 15;19:36. doi: 10.1186/s12989-022-00471-0 (PMC9107651; doi:10.1186/s12989-022-00471-0)
Supplement: Supplementary file 5 — Additional file 5: Table S2. Average Metal Exposure to Security Forces (all values in µg/m³). [file 12989_2022_471_MOESM5_ESM.pptx]

## Slide 1
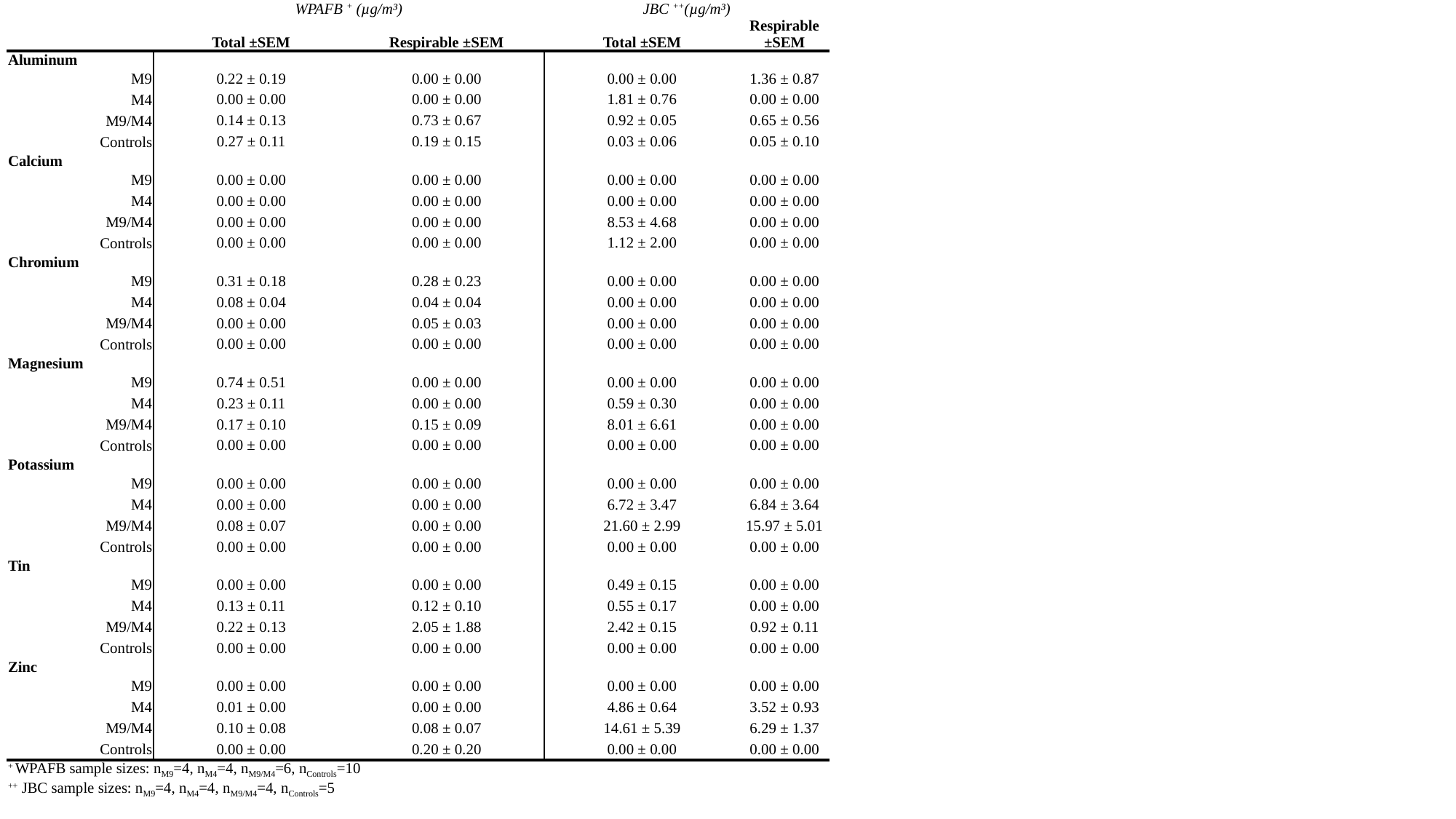

| | WPAFB + (µg/m³) | | JBC ++(µg/m³) | | |
| --- | --- | --- | --- | --- | --- |
| | Total ±SEM | Respirable ±SEM | Total ±SEM | Respirable ±SEM | |
| Aluminum | | | | | |
| M9 | 0.22 ± 0.19 | 0.00 ± 0.00 | 0.00 ± 0.00 | 1.36 ± 0.87 | |
| M4 | 0.00 ± 0.00 | 0.00 ± 0.00 | 1.81 ± 0.76 | 0.00 ± 0.00 | |
| M9/M4 | 0.14 ± 0.13 | 0.73 ± 0.67 | 0.92 ± 0.05 | 0.65 ± 0.56 | |
| Controls | 0.27 ± 0.11 | 0.19 ± 0.15 | 0.03 ± 0.06 | 0.05 ± 0.10 | |
| Calcium | | | | | |
| M9 | 0.00 ± 0.00 | 0.00 ± 0.00 | 0.00 ± 0.00 | 0.00 ± 0.00 | |
| M4 | 0.00 ± 0.00 | 0.00 ± 0.00 | 0.00 ± 0.00 | 0.00 ± 0.00 | |
| M9/M4 | 0.00 ± 0.00 | 0.00 ± 0.00 | 8.53 ± 4.68 | 0.00 ± 0.00 | |
| Controls | 0.00 ± 0.00 | 0.00 ± 0.00 | 1.12 ± 2.00 | 0.00 ± 0.00 | |
| Chromium | | | | | |
| M9 | 0.31 ± 0.18 | 0.28 ± 0.23 | 0.00 ± 0.00 | 0.00 ± 0.00 | |
| M4 | 0.08 ± 0.04 | 0.04 ± 0.04 | 0.00 ± 0.00 | 0.00 ± 0.00 | |
| M9/M4 | 0.00 ± 0.00 | 0.05 ± 0.03 | 0.00 ± 0.00 | 0.00 ± 0.00 | |
| Controls | 0.00 ± 0.00 | 0.00 ± 0.00 | 0.00 ± 0.00 | 0.00 ± 0.00 | |
| Magnesium | | | | | |
| M9 | 0.74 ± 0.51 | 0.00 ± 0.00 | 0.00 ± 0.00 | 0.00 ± 0.00 | |
| M4 | 0.23 ± 0.11 | 0.00 ± 0.00 | 0.59 ± 0.30 | 0.00 ± 0.00 | |
| M9/M4 | 0.17 ± 0.10 | 0.15 ± 0.09 | 8.01 ± 6.61 | 0.00 ± 0.00 | |
| Controls | 0.00 ± 0.00 | 0.00 ± 0.00 | 0.00 ± 0.00 | 0.00 ± 0.00 | |
| Potassium | | | | | |
| M9 | 0.00 ± 0.00 | 0.00 ± 0.00 | 0.00 ± 0.00 | 0.00 ± 0.00 | |
| M4 | 0.00 ± 0.00 | 0.00 ± 0.00 | 6.72 ± 3.47 | 6.84 ± 3.64 | |
| M9/M4 | 0.08 ± 0.07 | 0.00 ± 0.00 | 21.60 ± 2.99 | 15.97 ± 5.01 | |
| Controls | 0.00 ± 0.00 | 0.00 ± 0.00 | 0.00 ± 0.00 | 0.00 ± 0.00 | |
| Tin | | | | | |
| M9 | 0.00 ± 0.00 | 0.00 ± 0.00 | 0.49 ± 0.15 | 0.00 ± 0.00 | |
| M4 | 0.13 ± 0.11 | 0.12 ± 0.10 | 0.55 ± 0.17 | 0.00 ± 0.00 | |
| M9/M4 | 0.22 ± 0.13 | 2.05 ± 1.88 | 2.42 ± 0.15 | 0.92 ± 0.11 | |
| Controls | 0.00 ± 0.00 | 0.00 ± 0.00 | 0.00 ± 0.00 | 0.00 ± 0.00 | |
| Zinc | | | | | |
| M9 | 0.00 ± 0.00 | 0.00 ± 0.00 | 0.00 ± 0.00 | 0.00 ± 0.00 | |
| M4 | 0.01 ± 0.00 | 0.00 ± 0.00 | 4.86 ± 0.64 | 3.52 ± 0.93 | |
| M9/M4 | 0.10 ± 0.08 | 0.08 ± 0.07 | 14.61 ± 5.39 | 6.29 ± 1.37 | |
| Controls | 0.00 ± 0.00 | 0.20 ± 0.20 | 0.00 ± 0.00 | 0.00 ± 0.00 | |
| + WPAFB sample sizes: nM9=4, nM4=4, nM9/M4=6, nControls=10 | | | | | |
| ++ JBC sample sizes: nM9=4, nM4=4, nM9/M4=4, nControls=5 | | | | | |
