# Supplementary material for: Exposure to lead-free frangible firing emissions containing copper and ultrafine particulates leads to increased oxidative stress in firing range instructors
Source: Part Fibre Toxicol. 2022 May 15;19:36. doi: 10.1186/s12989-022-00471-0 (PMC9107651; doi:10.1186/s12989-022-00471-0)
Supplement: Supplementary file 6 — Additional file 6: Table S3. Multiple linear regression model for urinary 8-OHdG level estimations. [file 12989_2022_471_MOESM6_ESM.pptx]

## Slide 1
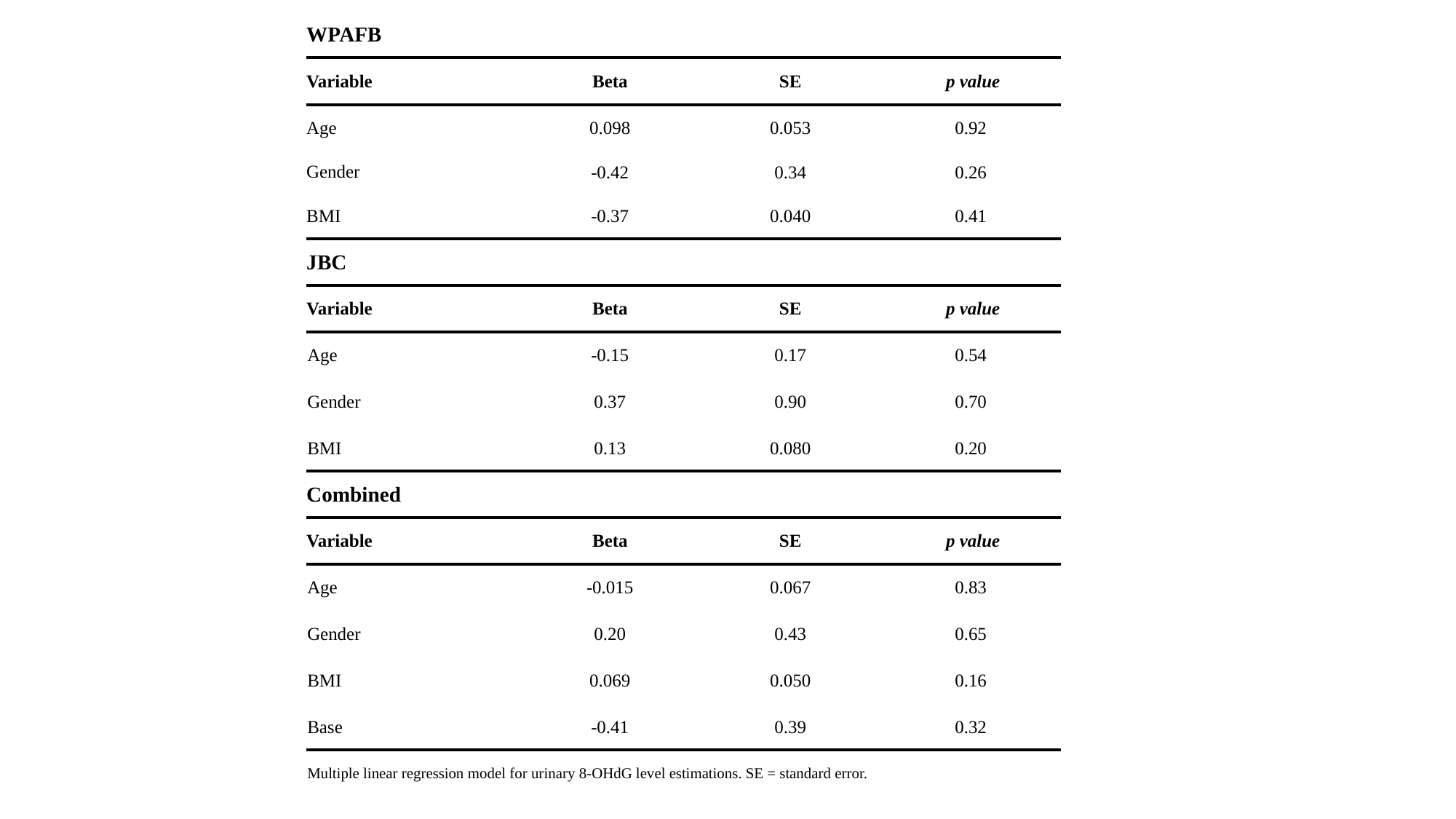

| WPAFB | | | |
| --- | --- | --- | --- |
| Variable | Beta | SE | p value |
| Age | 0.098 | 0.053 | 0.92 |
| Gender | -0.42 | 0.34 | 0.26 |
| BMI | -0.37 | 0.040 | 0.41 |
| JBC | | | |
| Variable | Beta | SE | p value |
| Age | -0.15 | 0.17 | 0.54 |
| Gender | 0.37 | 0.90 | 0.70 |
| BMI | 0.13 | 0.080 | 0.20 |
| Combined | | | |
| Variable | Beta | SE | p value |
| Age | -0.015 | 0.067 | 0.83 |
| Gender | 0.20 | 0.43 | 0.65 |
| BMI | 0.069 | 0.050 | 0.16 |
| Base | -0.41 | 0.39 | 0.32 |
| Multiple linear regression model for urinary 8-OHdG level estimations. SE = standard error. | | | |
