# Supplementary material for: Exposure to lead-free frangible firing emissions containing copper and ultrafine particulates leads to increased oxidative stress in firing range instructors
Source: Part Fibre Toxicol. 2022 May 15;19:36. doi: 10.1186/s12989-022-00471-0 (PMC9107651; doi:10.1186/s12989-022-00471-0)
Supplement: Supplementary file 7 — Additional file 7: Table S4. Multiple linear regression model for urinary Cu level estimations. [file 12989_2022_471_MOESM7_ESM.pptx]

## Slide 1
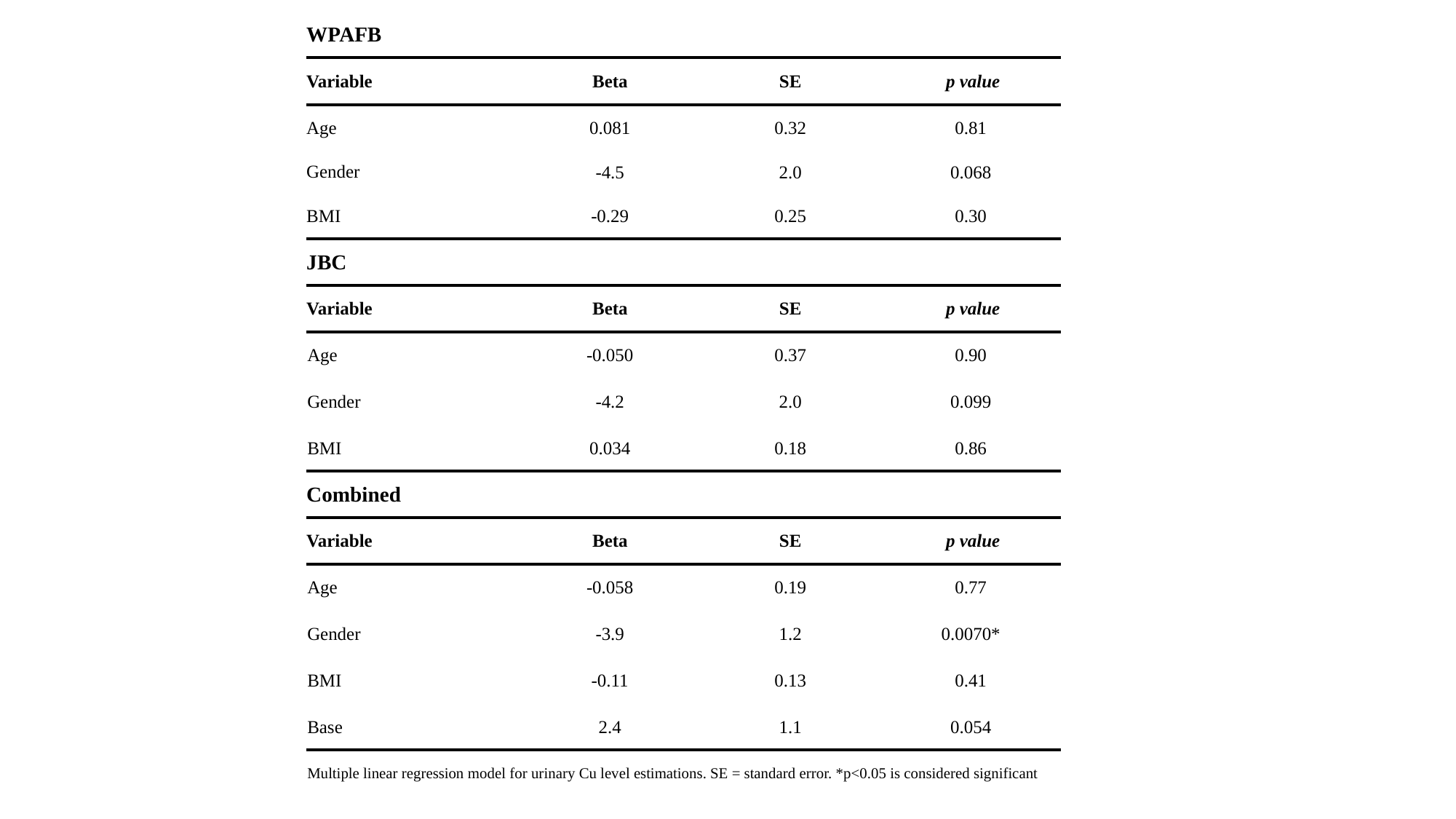

| WPAFB | | | |
| --- | --- | --- | --- |
| Variable | Beta | SE | p value |
| Age | 0.081 | 0.32 | 0.81 |
| Gender | -4.5 | 2.0 | 0.068 |
| BMI | -0.29 | 0.25 | 0.30 |
| JBC | | | |
| Variable | Beta | SE | p value |
| Age | -0.050 | 0.37 | 0.90 |
| Gender | -4.2 | 2.0 | 0.099 |
| BMI | 0.034 | 0.18 | 0.86 |
| Combined | | | |
| Variable | Beta | SE | p value |
| Age | -0.058 | 0.19 | 0.77 |
| Gender | -3.9 | 1.2 | 0.0070\* |
| BMI | -0.11 | 0.13 | 0.41 |
| Base | 2.4 | 1.1 | 0.054 |
| Multiple linear regression model for urinary Cu level estimations. SE = standard error. \*p<0.05 is considered significant | | | |
